# Drivers of seasonal dynamics in *Ulva* spp. associated microbiota and surface metabolome: The interplay between environment and host physiology

**DOI:** 10.1016/j.crmicr.2025.100439

**Published:** 2025-07-11

**Authors:** Sauvann Paulino, Cyril Noël, Laura Rieusset, Laure Taupin, Gwenaelle Le Blay, Nathalie Bourgougnon

**Affiliations:** a UBS, Laboratoire de Biotechnologie et Chimie Marines, EMR CNRS 6076, Vannes/Lorient, France; b Univ Brest, CNRS, IRD, Ifremer, LEMAR, F-29290 Plouzané, France; c IFREMER-IRSI-Service de Bioinformatique (SeBiMER), Plouzané, France

**Keywords:** Epiphytic bacteria, Surface metabolome, Temporal dynamic, Seasonal drivers

## Abstract

Marine ecosystems are increasingly affected by climate change and eutrophication, placing considerable stress on macroalgae and potentially disrupting their mutualistic interactions with surface-associated microorganisms, especially bacteria. This study explored the temporal dynamics of epiphytic microbial communities (16S rRNA) and the surface metabolome (LC-MS) of *Ulva* spp. from southern Brittany (France) over a 17-month period. The data obtained were analyzed through multivariate techniques to uncover correlations between bacterial community structure and surface metabolites. Biochemical and environmental parameters were also examined to identify the factors driving temporal variations in the macroalgal surface landscape. The analysis revealed distinct bacterial communities on *Ulva* spp. surface, compared to those in surrounding seawater or on rocky substrate. Key environmental factors, including salinity, nitrate concentration, and precipitation, as well as the physiological traits of *Ulva* spp. (such as uronic acid content, lipid levels, and moisture), were identified as primary drivers of microbiota dynamics. A stable core microbial community, primarily composed of *Alphaproteobacteria, Flavobacteriia, Oligoflexia*, and *Saprospira*, was also observed within the *Ulva* spp. microbiota. Finally, correlations between Amplicon Sequence Variants (ASVs) and surface metabolites underscored the importance of an integrated multi-omics approach to enhance our understanding of the complex dynamics between eukaryotic hosts and their associated microbiota.

## Highlights

• Temporal monitoring of the microbiota and surface metabolome of *Ulva* spp.
• Active selection of bacteria composing the microbiota on the surface of *Ulva* spp.
• Impact of seasonal factors on the microbiota and surface metabolome of *Ulva* spp.
• Link between the physiological state of the algal host and the dynamics of the microbiota.

## Graphical abstract

**Figure:**
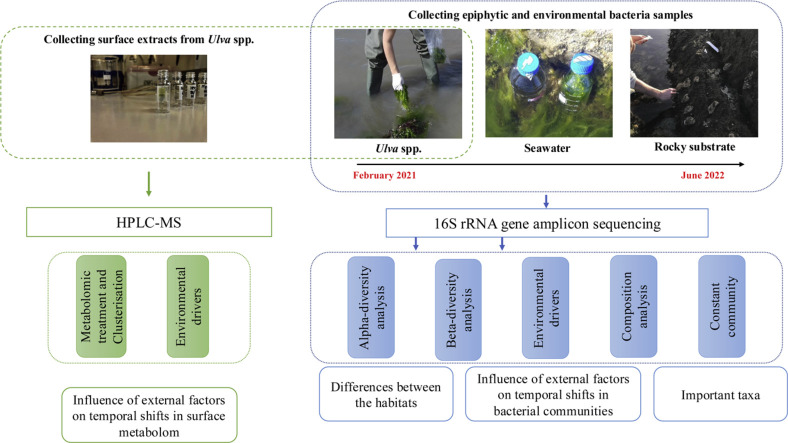

## 1 Introduction

Macroalgae play a pivotal role in marine ecosystems by producing oxygen, providing shelter for diverse marine species, and serving as a food source for a wide range of herbivores (Pfister et al., 2019; García-Poza *et al*., 2022). However, macroalgal-based ecosystems are facing growing threats from human activities, such as coastal development, pollution, and industrial practices, as well as from climate change-related factors, including rising temperatures, ocean acidification, sea-level rise, and the spread of invasive species (Filbee-Dexter *et al*., 2020; Pacheco *et al*., 2020; García-Poza *et al*., 2022). Over time, these disturbances may drastically alter the biogeographic distribution of macroalgae and compromise the critical ecosystem services they provide.

Growing interest has been directed toward understanding the interactions between macroalgae and their associated microorganisms. As with all submerged surfaces in seawater, macroalgae are colonized by diverse microbial communities. The composition of these communities is influenced by factors such as the section and age of the thallus, the season, the location, and the algal species (Jensen et al., 1996; Green and Bohannan, 2006; Trias *et al*., 2012; Egan *et al*., 2013; Goecke *et al*., 2013; Nemergut et al., 2013). The concept of the holobiont, originally developed for corals (Rosenberg *et al*., 2007; Barott *et al*., 2011), is highly relevant for macroalgae, as surface-associated microbial communities play vital roles in various aspects of the algal life cycle (Singh and Reddy, 2014; Egan et al., 2013) and are functionally integrated with their eukaryotic host.

Environmental stressors, however, can destabilize these symbiotic relationships, resulting in a loss of epiphytic bacteria and a breakdown of the holobiont, a phenomenon known as holobiont break-up (De Fouw et al., 2016; Egan et al., 2017). This disruption often leads to dysbiosis, characterized by microbial imbalance, which can negatively impact algal health and ecosystem stability (Campbell, 2011; Fernandes *et al*., 2012; Zozaya-Valdes *et al*., 2015).

*Ulva,* a genus of green macroalgae, has emerged as a model for studying macroalgae-bacteria interactions. Its associated bacterial community is known to play essential roles in reproduction, growth, and defense. For example, bacteria associated with *Ulva* promote reproduction by attracting zoospores for adhesion (Joint *et al*., 2002; 2007), support growth through the release of morphogenetic factors such as thallusin (Matsuo *et al*., 2005; Spoerner *et al*., 2012; Alsufyani *et al*., 2020), and contribute to defense due to their antibacterial properties (Ismail et al., 2018). Furthermore, *Ulva mutabilis* has become a model organism for exploring macroalgal growth and developing a reductionist model of bacteria-induced morphogenesis (Wichard, 2023). Unlike many macroalgae, *Ulva* can benefit from anthropogenic environmental stressors, proliferating under eutrophic conditions and causing green tides in coastal regions worldwide (Ye *et al*., 2011).

Fluctuations in *Ulva*'s microbiota across environmental gradients raise important questions about the stability of its bacterial community over time and under varying environmental conditions. Understanding this stability is crucial in the context of global change, as it informs efforts to define what constitutes a "healthy" microbial community (Risely, 2020) and improves the sustainability of macroalgal aquaculture (Califano *et al*., 2020). However, studies on *Ulva*'s core microbiota remain inconclusive. While some have reported bacterial taxa that persist across spatial scales (Tujula *et al*., 2010; Liu *et al*., 2023; Van der Loos *et al*., 2023), longitudinal gradients (Van der Loos *et al*., 2023), or temporal monitoring (Comba González et al., 2021), others emphasize bacterial assemblages based on functional roles (Burke, Steinberg, *et al*., 2011; Roth-Schulze *et al*., 2018).

Moreover, although seasonal and environmental variations in *Ulva*'s surface-associated bacterial communities have been documented (Lachnit *et al*., 2011; Comba González et al., 2021; Van der Loos et al., 2023), no in situ study has simultaneously monitored the microbiota and metabolome of *Ulva* over time. Such an approach, while studied under experimental conditions as part of reductionist model systems, remains largely unexplored in natural settings.

The aim of this study was to investigate the temporal variations in the epibacterial community and surface metabolome of *Ulva* spp. along a northwestern Atlantic shore in France, while accounting for environmental and physiological parameters. Metabarcoding analyses were conducted to characterize the specificity of the bacterial communities on the *Ulva* spp. surface and identify portions of the community that remained relatively stable over time. To evaluate this specificity, bacterial communities from surrounding seawater and biofilm on rocky substrates near the algal specimens were also sampled. Due to the limited taxonomic resolution inherent to short-read amplicon sequencing, particularly at the ASV or species level, we chose to focus our analysis at the genus level—a more reliable and interpretable taxonomic scale for ecological inference. This approach aligns with previous studies on algal–bacterial interactions and is especially relevant for formulating functional hypotheses, as many traits of interest—such as biofilm formation (Wahl *et al*., 2012) or algal polysaccharide degradation (Martin *et al*., 2014) are conserved at the genus level. Environmental parameters were also thoroughly assessed to identify their contributions to temporal variation in microbiota composition. Adopting this taxonomic and analytical framework allowed us to investigate microbiota–metabolome–environment relationships in a robust and comparable manner across both spatial and temporal gradients.

By combining meta-omics approaches, this study advances our knowledge of the complexity and stability of macroalgal-microbial interactions in the context of global environmental change.

## 2 Experimental procedures

### 2.1 Sampling

The surface microbiota of the algae, the rocky substrate and the seawater were monitored from February 2021 to June 2022 every other month. The sampling site was located in Kerleven beach along three stations in the southern coast of Finistere (France): station 1 (47°53′36.69′'N; 3°58′16.9′'W) with a direct access to the beach, and stations 2 (47°53′24.95′'N; 3°57′24.95′'W) and 3 (47°53′40′'N; 3°57′22.78′'W), two infralittoral stations only discovered during low tides with high coefficients (>80). *Ulva* spp. thalli were collected at high-coefficient low tide by carefully detaching from rocky substrate with sterile gloves and immediately stored in sterile bottles filled with surrounding seawater. For each station, three different individuals collected nearby were considered as three biological replicates. One individual per station was also swabbed on site in June 2022 to demonstrate that sample transport did not impact the microbiota structure. In addition to algal samples, surrounding water (1 L in triplicate) was collected in sterile bottles and rocky substrates were swabbed, in triplicate. The rocky substrate swabs (Ozyme, France) were immediately put into DNA/RNA Shield Collection Tubes (Zymo Research, Irvine, CA, USA) containing an extraction and stabilizing buffer and placed at −20 °C upon arrival at the laboratory. From the point of collection, samples were transported in cool boxes to maintain the seawater temperature.

Samples were processed in the laboratory within 2 h. Upon arrival at the laboratory, *Ulva* spp. samples were rinsed three times with sterile artificial seawater (ASW, Sea salt 30 g.L^-1^, Sigma-Aldrich, St. Louis, USA). To ensure multi-omics cross-comparison, each thallus was separated into two equal parts for metabarcoding and metabolomic analyses.

#### 2.1.1 Characterization of the algal host

*Ulva* specimens collected between February 2021 and February 2022 were sent to Pr. de Clerck (Research group phycology, Ghent University) for molecular identification based on the *tufA* gene sequencing, according to the protocol described by Van der Loos *et al*., 2023**.** Moreover, biochemical analyses were carried out to characterize the composition of the algae**.** Furthermore, the surface area of each alga was measured using the image J software, and the moisture content was determined by the ratio of wet and dry weight after 36 h in a laboratory oven (60 °C).

#### 2.1.2 Characterization of the environment

Seawater parameters (salinity, pH, fluorescence, depth, temperature, turbidity, dissolved oxygen) were measured with a multiparameter probe (YSI 6920 V2–2, A xylem brand). These parameters were collected in December 2021 and June 2022 to characterize the sampling stations in two contrasting seasons (**Supplementary Table 3**)**.**

Temporal monitoring of environmental data over 17 months was facilitated by datasets obtained from Previmer models (ECO-MARS3D, https://marc.ifremer.fr) and Météo France (Quimper station n°29,216,001, 47°58′22″N - 4°09′38″W). These datasets included atmospheric variables such as atmospheric temperature, solar radiation, irradiation duration, precipitation, relative humidity, and wind speed. In addition, surface seawater parameters were characterized, including temperature and salinity, as well as proxies for primary production, such as chlorophyll a, nitrates, and phosphates.

### 2.2 Surface metabolome extraction and analyses

Extraction of surface metabolomes were standardized to 45 mm diameter by using a cookie cutter. Each algal fragment was dipped in 2 mL of methanol or 2 mL of n-hexane for 20 s. This extraction procedure was performed independently on three replicate algal fragments per station, and the extracts for each solvent were then pooled. A total of 81 extracts from the surface metabolome of *Ulva* spp. were collected (9 per sampling date). In order to collect only surface metabolites, particular care was taken not to dip the algal fragments edges in solvents. Similarly, preliminary tests were done to identify dipping times that would not damage the algal cell wall. Surface extracts were concentrated by evaporating the solvent with a vacuum concentrator (Genevac™, United-Kingdom). They were then stored in sealed vials at −20 °C until analysis.

For LC-MS analyses, samples were prepared by solubilizing the dried extracts in a 200 μL mixture of acetonitrile (ACN) and water (H_2_O) (65:35, v/v) acidified with formic acid (0.1 %). Additionally, one experimental blank was prepared and injected at the beginning and at the end of the injection sequence. The chemical profiling of the surface extracts was obtained using an LC-ESI-MS (Dionex, Ultimate 3000 Bruker, Germany) coupled with a QToF Impact II mass spectrometer (Bruker Daltonik GmbH, Germany) in positive ionization mode. Separations were carried out with an analytical reverse-phase column. A 10 μL extract sample was injected for each analysis, at a flow rate of 0.5 mL.min^-1^ with a column temperature of 30 °C. In order to optimize the surface extract signal, two injection heights were defined in order to analyze the ACN phase and the aqueous phase separately. The ACN phase proved to be the most convincing in terms of providing defined peaks. The elution gradient was adopted from Othmani *et al*. (2016). The gradient set up was as follows: (i) an initial isocratic stage of 10 min with ACN/H2O (65:35, v/v); (ii) a linear gradient passing from 65 % to 100 % ACN in 20 min; (iii) an isocratic stage of 30 min with 100 % ACN, finishing by a return to initial conditions (0.1 min) and equilibration of the column (9.9 min). Regarding the mass spectrometer, operating conditions were set as follow: drying temperature: 350 °C, capillary voltage: 4 kV, nebulizer pressure: 3.45 bar, drying gas: helium at a flow rate of 12 L min−1 (Othmani et al. 2016). Mass spectra acquisition was set at 0.5 Hz from *m/z* 50 to 1000.

### 2.3 Pre-processing of metabolomic data

Eighty-one LC/MS data were exported in .mzXML format and pre-processed by MZmine 4.3.0 for peaks searching. The parameters used for the analyses were inspired by those used by Remy et al. (2019). Blanks were analyzed to define a minimum noise threshold. Mass detection was performed using a centroid algorithm with a noise level threshold set at 5000. The retention time considered was set between 3 and 21 min. Chromatogram formation (ADAP chromatogram builder) was established using a minimum scan group size of 4, a group intensity threshold of 3000, a minimum intensity of the highest data of 4000 and an *m/z* tolerance of 0.05 (or 20 ppm). The ADAP wavelet deconvolution algorithm was used with the following parameters: S/N threshold of 8, minimum peak height = 4000, area coefficient/threshold = 2, peak duration range = 0.001–2 min, wavelet range = 0.001 - 1 min. The list of identified peaks was aligned using the joint aligner algorithm with a *m/z* tolerance of 0.01 (or 50 ppm) and a retention time tolerance of 0.5 min, weight for *m/z*= 1 for retention time = 1. The results were exported in *.csv format and included all the features detected, referenced by their *m/z* and retention time (rt, minutes), as well as their intensity. The final matrix corresponded to a total of 358 characteristic features (*m/z*; rt) present in at least 10 among 81 samples as well as their respective intensity, transformed by a log-10, for the 81 samples investigated. The metabolomics data were first analyzed by Principal Component Analysis (PCA) to determine whether there was a separation of *Ulva* spp. surface extracts according to the month of sampling and environmental factors, and then integrated into the multi-omics analysis as response variables.

### 2.4 DNA extraction, 16S rRNA gene amplification and sequencing

Upon arrival at the laboratory, each thallus was thoroughly rinsed with sterile artificial seawater to remove non-surface-associated bacteria. The surface of each of the six fragments composing a statistical individual was then gently swabbed over an area of approximately 10–12 cm² under sterile conditions inside a microbiological safety cabinet (MSC). In parallel, rocky biofilm samples were swabbed directly in situ over the same surface area (∼10–12 cm²). Concurrently, one litre of seawater (in triplicate) was filtered through polycarbonate membranes (0.22 µm, Whatman), which were immediately placed in cryotubes and flash frozen in liquid nitrogen before storage at −80 °C. DNA from epiphytic bacteria was extracted from the swabs according to the ZymoBIOMICS DNA/RNA Mini Kit (Zymo Research) protocol. Rocky substrate and seawater DNA were extracted using a PCI extraction as described in Offret et al. (2020). DNA concentrations from the algal surface, seawater and rocky substrate were quantified using spectrofluorometry with the Quantifluor kit (Invitrogen, Massachusetts, USA) and a NanoDrop^Tm^ Spectrophotometer (Ozyme, France). Bacterial diversity was investigated by targeting the V3-V4 region of the 16S rRNA gene, using the NOCHL primers developed by Thomas et al. (2020) to minimize plastid amplification. This set of primers consisted of the forward primer S-d-Bact-0341-b-S-17 (CCTACGGGNGGCWGCAG), and the reverse primer S-d-Bact-0785-a-A-21 (GACTACHVGGGTATCTAATCC), and amplified a 478 bp fragment (Thomas et al.*,* 2020). The amplicons were paired-end sequenced (2 × 250 bp) on an Illumina MiSeq platform (Genome Québec, Canada). Sequences assessed in this study have been submitted to European Nucleotide Archive (ENA) under the accession number PRJEB85979.

### 2.5 Metabarcoding data processing and statistical analyses

Raw data were analyzed using the SAMBA v3.0.1 workflow (Noël et al., 2021; https://github.com/ifremer-bioinformatics/samba) developed by the SeBiMER (Ifremer’s Bioinformatics Core Facility), a Standardized and Automatized MetaBarcoding Analysis workflow using DADA2 (Callahan et al.*,* 2016) and QIIME2 (Bolyen *et al*., 2019) with default parameters unless otherwise indicated. A first step of raw data integrity checking was performed after sequencing with the SAMBA checking process. Then, sequencing primers were trimmed from reads and reads without primers were removed. Afterward, DADA2 was used to filter poor quality reads, correct sequencing errors as well as overlap paired reads, infer Amplicon Sequence Variants (ASVs) and remove chimeras. A step of ASV clustering was added using the dbOTU3 algorithm (Olesen et al., 2017) relying on both distribution of ASVs and phylogenetic distance, to correct the diversity overestimation generated by the ASV inference from DADA2. Finally, ASV taxonomy was assigned using a Naïve Bayes classifier against the SSU SILVA (Version 138) database (Pruesse et al. (2007); https://www.arb-silva.de). Consequently, the names assigned to the sequences correspond to the classification based on the SILVA v138.1 database (Quast et al., 2012). In total, 8593,157 reads were generated with 38,023 average per sample obtained through the sequencing of 84 algal surface samples, 81 seawater samples and 63 rocky substrate samples corresponding to 13,619 ASVs. Rarefaction curves showed a sufficient sequencing effort to describe the bacterial diversity among the different habitats (**Supplementary Figure 1**)**.** A total of 211 samples with an abundance of at least 0.01 % of the total abundance were retained for further analyses (Alberdi et al., 2018). Alpha-diversity metrics (Chao1, Shannon and invSimpson indexes, Pielou evenness) were calculated using the Phyloseq R package and tested statistically with a T-test followed by a post hoc test (Bonferroni multiple-significance-test correction).

A core taxa was identified at the genus level by grouping ASVs assigned to the same genus. A genus was considered part of the core microbiota if it was detected in at least one replicate per sampling station (3 stations) for each of the 9 sampling months (see Paix et al.*,* 2019). Prior to beta-diversity analysis, data were normalized using the ‘cumNormStatFast’ function with metagenomeSeq R package (Paulson, 2013) performing a cumulative-sum scaling (CSS). Dissimilarity matrices using the Weighted-UniFrac distances were represented in a two-dimensional space by Non-metric multidimensional scalings (nMDS). The similarity differences observed between the three habitats and between each month within the nMDS, was statistically tested with a PERMANOVA test (999 permutations) using the Vegan R package. Then, environmental and biochemical variables were overlaid onto the Bray Curtis based-distance nMDS plots without disrupting the configuration of the original ordinations using function ‘envfit’ from the Vegan R package. A R^2^ measure of fit and a “significance” value based on the probability that random permutations of the environmental variables (permutations = 999) would yield a higher degree of fit than the true environmental variables were also generated. Finally, predictive cellular functions, which were likely associated with a marker gene based on its sequence similarity with a reference genome, were inferred using the PICRUSt2 tool (Douglas *et al*., 2020).

### 2.6 Integration of metabolomic, metabarcoding, and predicted functions dataset

Metabarcoding and predicted functions datasets were filtered to remove features for which the sum of counts were below a certain threshold (removing ASVs/features for which the sum of counts are below 1 % of the total sum of all counts, see Arumugam et al. (2011). A Log-transformation was then performed on those two datasets; the metabolomic dataset had already been filtered and Log-transformed in previous metabolomic treatment. A correlation analysis between the three datasets was performed with the multiblock sPLS-DA method (referred to as DIABLO), used to perform a PLS Discriminant Analysis (PLS-DA) on more than two datasets, from MixOmics R package (Lê Cao et al., 2008). The purpose of this multivariate analysis was to perform an N-block integration of different omic datasets to identify the most correlated ASVs, metabolites and predicted functions involved in the discrimination of each month. A correlation circle plot was output to exhibit selected features on a circle and the links between or within omic features, which presented strong positive or negative correlations.

## 3 Results

### 3.1 Characterization of the algal host and the environment

**Fig. 1:**
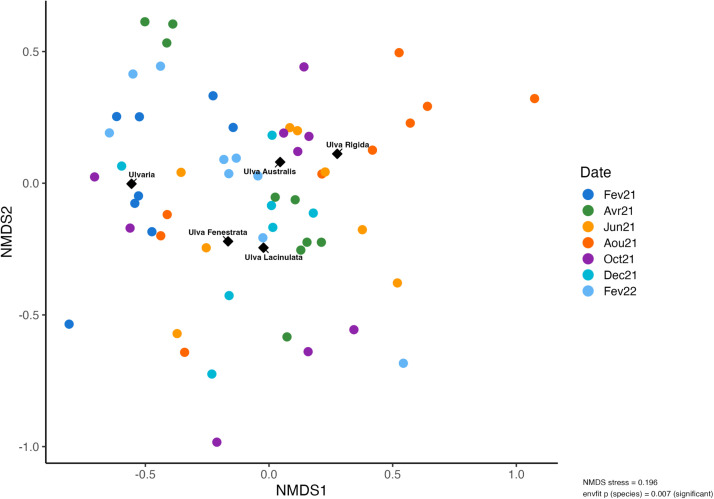
Non-metric multidimensional scaling of Bray-Curtis distance computed from CSS-transformed ASV abundances for the Ulva-associated bacterial community across all stations (S1, S2, S3) and replicates, sampled between February 2021 and February 2022 (Stress: 0.19). Host species were included as explanatory variables using ‘envfit’ with centroid positions displayed.

Molecular identification based on *tufA* gene sequencing revealed the presence of four distinct species within the *Ulva* genus across stations 1, 2, and 3 between February 2021 and February 2022 (**Supplementary Figure 2**). *Ulva australis* emerged as the dominant species, consistently detected throughout the sampling period (**Supplementary Table 1**)**.** Additionally, a representative of the genus *Ulvaria* was identified at station 2 in February 2021. Alpha diversity indices showed no significant differences in microbial richness and evenness among the various *Ulva* species and *Ulvaria* (**Supplementary Figure 3**). However, the macroalgal species factor had a significant effect on microbiota composition, as revealed by the *envfit* analysis (*p*< 0.05), indicating that host identity contributed to shaping epiphytic bacterial communities (Fig. 1)**.**

Despite this taxonomic heterogeneity, we retained all specimens in the microbial analyses, as they represented valuable replicates spanning environmental gradients and contributed to the overall diversity of the epiphytic community**.** This inclusive approach enabled a more comprehensive assessment of microbiota dynamics in situ. Biochemical analyses of specimens collected between June 2021 and June 2022 revealed significant monthly variations in the overall physiological state of the macroalgae (ANOVA, *p*< 0.05) (**Supplementary Figure 4 and Table 2**)**.** As anticipated, lipid content was significantly higher during winter (December/February 2021), while a notable decrease in total protein content was observed in April/June 2022. Neutral sugar levels increased markedly during the summer months (June 2021 and 2022, August 2021), whereas uronic acid content peaked primarily in April 2022 (post hoc test, Tukey's HSD, *p*< 0.05). In contrast, no clear patterns were observed in pigment assays. These biochemical data were subsequently integrated into analyses aimed at identifying the driving factors behind the temporal dynamics of bacterial communities associated with the surface of *Ulva* spp.

Results for the sampling site parameters are described in the Supplementary Materials, including the environmental data obtained from the Previmer models and Météo France, which were used in subsequent analyses (**Supplementary Tables 3 and 4**)**.**

### 3.2 Structure and diversity of epiphytic bacterial communities

Alpha-diversity was measured using Chao1, InvSimpson, Shannon and Pielou evenness indexes (**Supplementary Figure 5**)**.** When considering the bacterial communities associated with the three different habitats, all indexes showed significantly higher values (*p*< 0.05) for the biofilm samples collected on rocky substrates in comparison with the seawater and algal samples, which were not different from each other. Moreover, Pielou evenness index was above 0.7 for the three habitats (0.92 for rocky substrate), indicating an even distribution of ASVs within each bacterial community. No significant difference was observed between the sampling dates nor the sampling station within each habitat.

**Fig. 2:**
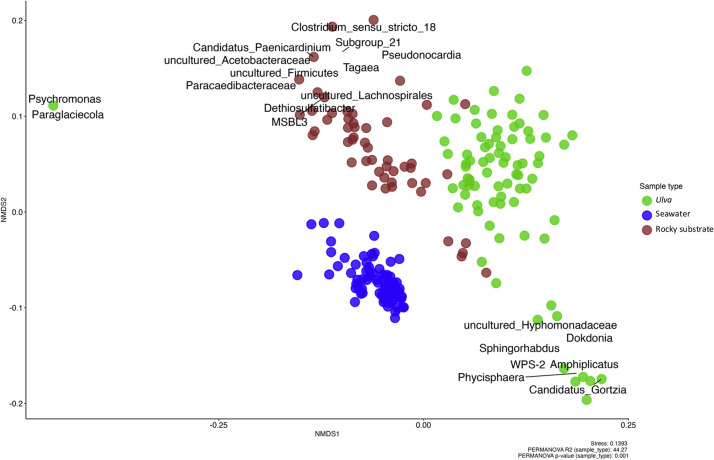
Non-metric multidimensional scaling of Bray Curtis distance computed from CSS-transformed ASVs abundances for the bacterial community of Ulva spp. (green), rocky substrate (red) and seawater (blue) sampled between February 2021 and June 2022 (Stress: 0.14).

Beta-diversity was analyzed using the weighted Unifrac distance matrix. As expected, the resulting NMDS plot (Fig. 2) showed a distinct clustering pattern between bacterial communities from surrounding seawater, rocky substrates and *Ulva* spp. surfaces. A greater proximity was observed between samples from the rocky substrate biofilm and the algal surface. In terms of dispersion, the seawater samples appeared less dispersed than those from the rocky substrate and the algal surface (betadisper, *p*< 0.05).

**Fig. 3:**
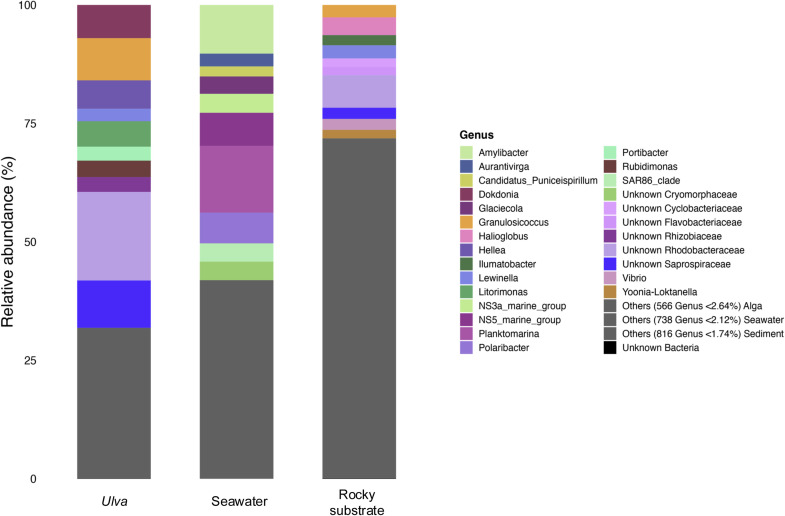
Relative abundance of majoritarian bacterial ASVs summarized at the genus taxonomic rank in associated communities of Ulva spp., seawater and rocky substrate.

The structure of the bacterial communities in the three habitats showed similarities at high taxonomic levels. At the class level, *Alphaproteobacteria* (>29 %), *Bacteroidia* (>26 %) and *Gammaproteobacteria* (>15 %) were the predominant groups (**Supplementary Figure 6**)**.** At the genus level, the structure of the bacterial communities was much more distinct between the three habitats (Fig. 3).

Within the algal samples, the genus *Granulosicoccus* and two unknown genera affiliated to the *Rhodobacteraceae* and *Saprospiraceae* families occupied a relative abundance over 8 % (with a relative abundance of 18.7 % for the unknown genus affiliated within the *Rhodobacteraceae*). In seawater samples, bacterial communities were dominated by *Amylibacter* and *Planktomarina* genera (*Alphaproteobacteria)*. In accordance with the high Pielou index, no genus dominated the rocky substrates samples, with almost 816 genera having a relative abundance of <1.74 %.

**Fig. 4:**
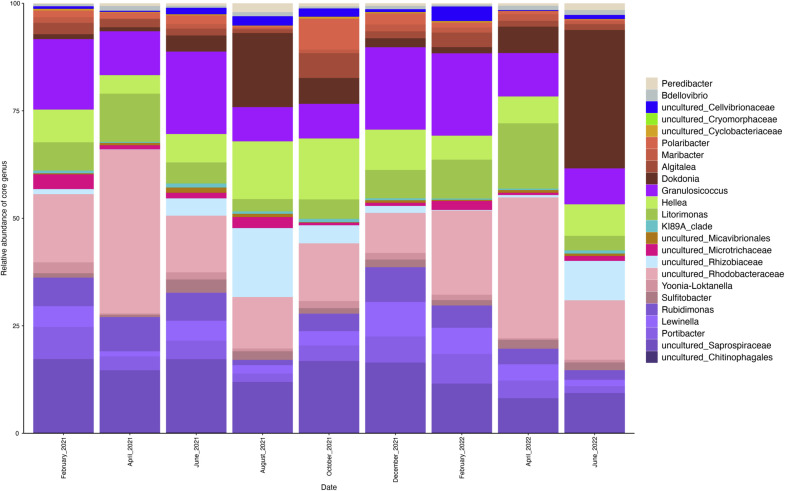
Relative abundance of ASVs belonging to the core Ulva spp. bacterial community between February 2021 and June 2022, summarized at the genera taxonomic rank.

Within the bacterial community associated with the algae, almost 50 % of the relative bacterial abundance was distributed among 10 genera. A bacterial community that remained relatively constant over time, that we named “core” was then identified. The 24 genera that make up this core community were present at all sampling stations for each of the nine sampling dates (Fig. 4).

These 24 genera belonged to the *Alphaproteobacteria, Flavobacteriia, Oligoflexia* and *Saprospiria* classes. However, the relative abundances of genera belonging to this core community associated with the algal surface varied over time. Indeed, the relative abundance of certain genera highly increased on certain dates. This was the case for the *Dokdonia*, which was particularly present in June 2022 (48 % of relative abundance) and to a lesser extent in August 2021 (21 %). Similarly, the uncultured *Rhizobiaceae* was predominant in summer, particularly between June 2021 and October 2021, with a relative abundance of almost 16 % in August 2021, as well as in June 2022. Similarities from one year to the next were also highlighted, notably for April 2021 and April 2022. The relative abundance of the uncultured *Rhodobacteraceae* occupied a large proportion of the stable algal community, reaching 39.4 % in April 2021 and 31.6 % in April 2022. In addition, the genus *Litorimonas* was, to a lesser extent, more present in April 2021 (12.4 %) and April 2022 (15 %). The core bacterial community constituted the majority on all nine sampling dates, with its proportion ranging from 59 % in February 2021 to 92.8 % in April 2021 and April 2022. The lower proportion observed in February 2021 was probably attributable to the high abundance of the genus *Paraglaciecola* (data not shown, not included in the core community) on that particular date.

Although we defined the core microbiota at the genus level, applying the same criteria at the ASV level revealed only a very limited number of shared ASVs across samples, reflecting the high taxonomic and compositional variability at finer resolution (**Supplementary Figure 7**)**.** Notably, 30 % of the total ASVs (1491) were affiliated with the 24 genera constituting the constant fraction of algal-associated bacteria, while the remaining 70 % were distributed among the 526 other genera, highlighting the diversity and transience of non-core taxa.

A constant bacterial community over time was also found in the seawater samples (**Supplementary Figure 8**)**.** This constant community was made up of 47 genera, 6 of which were in common with those associated with the algal surface (*Granulosicoccus, Hellea, Peredibacter, Polaribacter, Sulfitobacter, Yoonia*). These 47 genera belonged to the *Alphaproteobacteria, Gammaproteobacteria, Flavobacteriia, Acidimicrobiia, Cytophagia, Espilonproteobacteria, Fusobacteriia, Oligoflexia* and *Saprospiria* classes. Within *Alphaproteobacteria*, the dominant class in seawater samples, *Roseobacteraceae* was the majority family.

A comprehensive investigation was conducted on various environmental factors to explore the structuring of the bacterial communities colonizing the surface of the algae, the surrounding seawater and the rocky substrate to which the algae were attached. Although the study did not focus on the spatial distinction of the sampling stations, a divergence in the factors structuring the bacterial communities was observed between station 1 as compared to stations 2 and 3. Only samples from the two infralittoral pools (stations 2 and 3) were structured by the tested environmental drivers. Samples from station 1, located along the beach, were subjected to high anthropogenic influences (walk fishing, bathing), and appeared to be structured by unidentified drivers. Thus, this station was not included in the beta-diversity analysis.

**Fig. 5:**
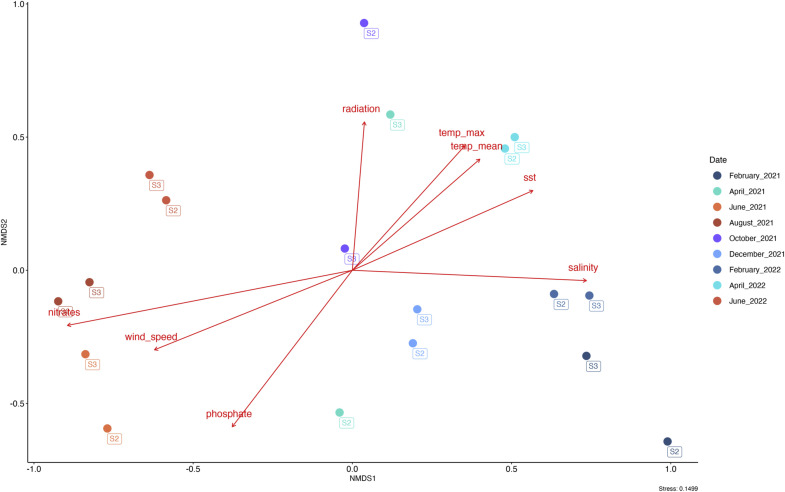
Non-metric multidimensional scaling of Bray Curtis distance computed from CSS-transformed ASVs abundances for the bacterial community in seawater sampled between February 2021 and June 2022 (Stress 0.15). Environmental parameters were added using envfit. The red arrows represent a statistically significant relationship (*p*> 0.05) measured through the envfit test.

Bacterial communities present in the surrounding seawater could be clustered in three distinct groups: ‘summer’ (June 2021, August 2021, June 2022), ‘mid-season’ (April 2021, October 2021, April 2022) and ‘winter’ (February 2021, December 2021, February 2022) communities [permutational multivariate analysis of variance (PERMANOVA); *p*< 0.05] (Fig. 5)**.**

The distinction between the clusters was influenced by various environmental factors. The ‘summer’ community was mainly driven by wind speed (*p*< 0.05, R^2^ = 48 %), nitrates (*p*< 0.001, R^2^ = 48 %) and phosphate concentrations (*p*< 0.05, R^2^ = 49 %). In summer, wind intensity was significantly weaker, while concentrations of nitrates and phosphates increased at the surface of the oceans. The intermediate community was structured by solar radiations (*p*< 0.1, R^2^ = 31 %), atmospheric temperatures (*p*≤ 0.05, R^2^ = 34 %) and sea surface temperatures (*p*< 0.05, R^2^ = 41 %). Finally, ‘winter’ community was mainly impacted by surface salinity (*p*< 0.01, R^2^ = 54 %), which slightly decreased in winter. No environmental drivers were highlighted to explain the dynamic of the rocky substrate bacterial communities.

**Fig. 6:**
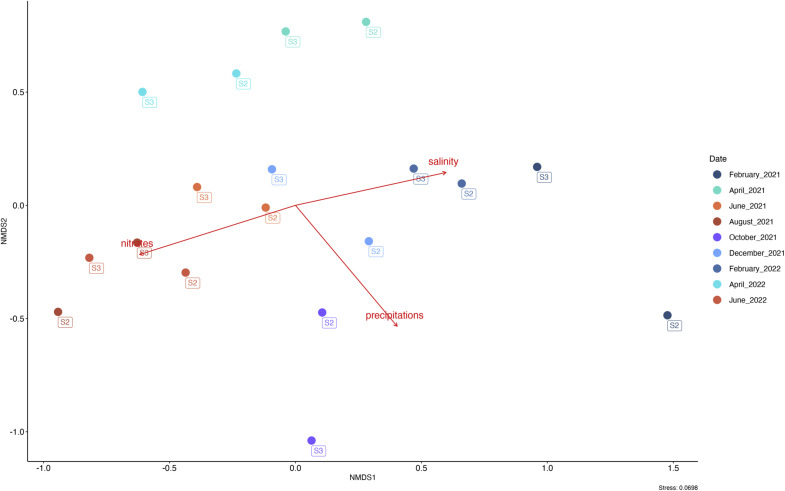
Non-metric multidimensional scaling of Bray Curtis distance computed from CSS-transformed ASVs abundances for the bacterial community of Ulva spp. sampled between February 2021 and June 2022 (Stress: 0.07). Environmental parameters were added using envfit.The red arrows represent a statistically significant relationship (*p*> 0.05) measured through the envfit test.

Epiphytic bacterial communities also varied according to both environmental parameters and the physiological state of the algae. For the epiphytic communities, the ‘summer’ community was correlated with an increase of seawater nitrates concentrations (*p*< 0.05, R^2^ = 42 %) and the ‘winter’ community by the slight decrease of surface salinity (*p*< 0.05, R^2^ = 27 %) (Fig. 6)**.** Moreover, precipitation amounts (*p*≤ 0.01, R^2^ = 44 %), which reached a maximum in October 2021, were found to drive the ‘fall’ community. The samples collected from one year to the next, in particular those from April 2021 and April 2022, were close.

**Fig. 7:**
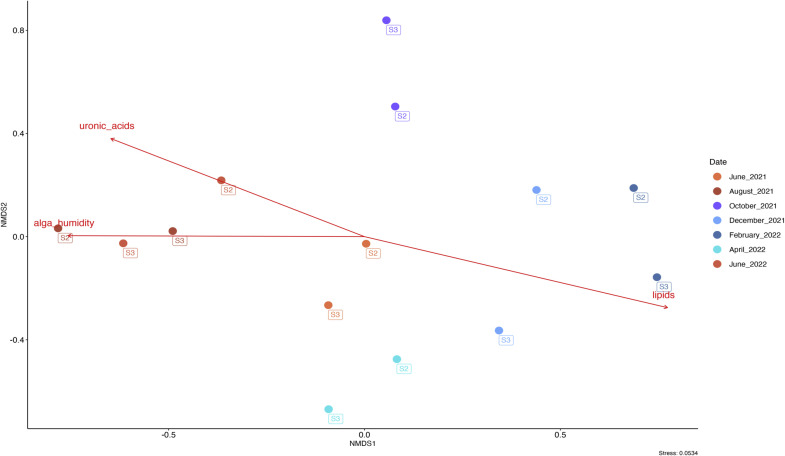
Non-metric multidimensional scaling of Bray Curtis distance computed from CSS-transformed ASVs abundances for the bacterial community of Ulva spp. sampled between June 2021 and February 2022 (Stress: 0.05). Biochemical parameters were added using envfit.The red arrows represent a statistically significant relationship (*p*> 0.05) measured through the envfit test.

A link was also established between the physiological state of the algae over the course of the year and its surface bacterial community (Fig. 7). The ‘summer’ bacterial community appeared to be correlated with increased uronic acid concentrations (*p*< 0.05, R^2^ = 56 %) and algal humidity (*p*< 0.01, R^2^ = 57 %), alongside a decrease in the algal lipid contents (*p*< 0.01, R^2^ = 67 %). In contrast, the ‘winter’ bacterial community was related to the increase in lipid content in algal tissues and the decrease in uronic acid concentrations.

### 3.3 Variation of the algal surface metabolites

**Fig. 8:**
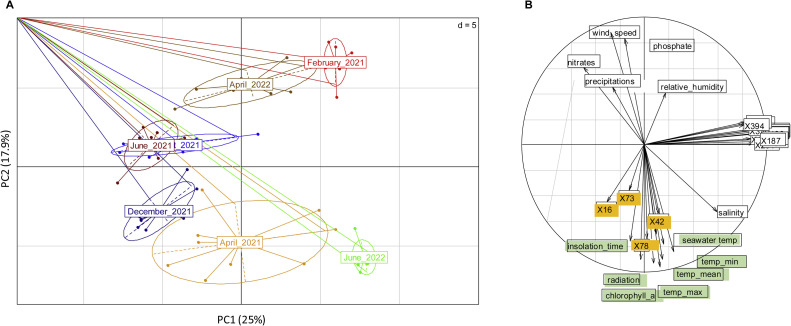
Principal component analysis of the seasonal structuring of the Ulva spp. surface metabolome (a); multivariate statistical analysis on discriminant features (orange) and abiotic parameters (b).

The surface extracts of *Ulva* spp. were analyzed from February 2021 to June 2022, resulting in the identification of 358 distinct features, each detected in at least 10 of the 81 samples analyzed. Two months, October 2021 and February 2021, displayed minimal features probably due to a problem during sample preparation, were therefore excluded from the analysis. First, principal component analysis performed on all the 358 features and 81 samples, clustered biological replicates from the same sampling month, thus demonstrating the consistency of the data (Fig. 8**a**)**.** Second, multivariate statistical analysis performed on discriminant compounds and abiotic parameters highlight relationship between environmental factors and four discriminant features (Fig. 8**b**)**.**

Features X16 (*m/z*: 114.09; retention time (min): 6.099); X73 (*m/z*: 451.17; retention time (min): 6.79); X78 (*m/z*: 80.95; retention time (min): 3.91) and X42 (*m/z*: 193.13; retention time (min): 8.44 were positively correlated (correlation > 0.75) with irradiation time, solar radiation, chlorophyll a concentration, atmospheric/seawater temperatures, salinity and negatively correlated with precipitation, nitrate and phosphate concentrations, wind speed and relative humidity. Among these five compounds, X16 was putatively identified as 5,5-dimethyl-1-pyrroline N-oxide (DMPO), while X42 was probably a chlorine molecule based on isotopic distribution.

### 3.4 Focus on sampling dates of interest

April 2021 and 2022 were very similar both in terms of surface bacterial structuration and chemical surface landscape. First, the stable bacterial community represented the majority (92.8 %) of total bacteria in April 2021 and 2022. Within this core community, *Rhodobacteraceae* occupied the largest part (39.4 % in April 2021 and 31.6 % in April 2022) of the surface bacterial community, leading to a high degree of similarity in April from one year to the next. Secondly, chemical surface profiles were very close, meaning that a large amount of the same metabolites were screened in April 2021 and April 2022 for algae sampled at the same locations. Finally, bacterial functions predicted on the basis of phylogeny (Picrust2), indicated similar functions in April 2021 and April 2022. Predictions were based on several gene family databases supported by the Kyoto Encyclopedia of Genes and Genomes11 (KEGG) orthologs (KOs) and Enzyme Commission numbers (EC numbers). Bacterial functions included production of phenylacetyl-CoA 1,2 epoxydase (pvalue < 0.001), proline dehydrogenase (pvalue < 0.05) and cyclic pyranopterin phosphate synthase (pvalue < 0.001). Metabolites synthesis (pvalue < 0.05) and Nicotinamide Adenine Dinucleotide (NAD) salvage pathway I (pvalue < 0.1) were also identified.

**Fig. 9:**
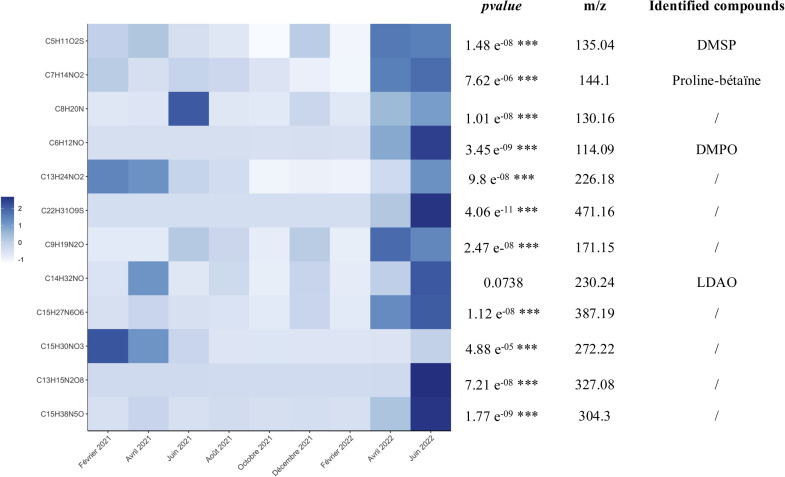
Heatmap of mean normalized and centered seasonal features intensity; p value < 0.05, Kruskall-Wallis test). Abbreviations: DMSP: diméthylsulfoniopropionate; DMPO: 5,5-Dimethyl-1-pyrroline N-oxide; LDAO: Lauryldimethylamine oxide.

Furthermore, June 2022 also presented a clearly distinct profile compared to the other dates. First, the surface extracts profile differed notably from the other periods, especially summer 2021 (June and August). Twelve metabolites, consistently detected across all extracts, were produced in significantly higher amounts in June 2022 compared to other months. These include metabolites identified using analytical standards, such as DiMethylSulfonioPropionate (DMSP), proline betaine, and LaurylDimethylAmine Oxide (LDAO), as well as putatively identified metabolites, including 5,5-DiMethyl-1-Pyrroline N—Oxide (DMPO) (Fig. 9)**.**

Among these 12 metabolites tested, 11 showed a significant difference from one month to the next (pvalue < 0.05, Kruskall Wallis test). This differentiation in the chemical profile on the algae surface in June 2022 was also mirrored in the composition of the associated bacterial community. Bacteria belonging to the genus *Dokdonia* were the most abundant, accounting for a total relative abundance of 22 % in June 2022. This relative abundance increased to 48 % when considering only the core bacterial community. Bacterial functions predicted also revealed the expression of peroxiredoxins in June 2022. On the other hand, considering the environmental data collected in June 2022, a maximum of solar radiation was recorded over the month (2062.833 Joules/cm², Météo France - Quimper, 47°58′22 "N - 4°09′38 "W). Moreover, Pearson's correlation tests indicated that solar radiation was significantly correlated with the increase of *Dokdonia* abundance (Coefficient = 0.56; *p*≤ 0.05). This could therefore be a potential environmental stress detected in June 2022, with a response from both the microbiota and the metabolome on the surface of *Ulva* spp.

### 3.5 Integration of surface metabolome, surface microbiota and putative bacterial functions datasets

Each dataset variable, encompassing surface metabolome, surface microbiota and putative bacterial functions, was integrated, and their correlations were assessed. The resulting vectors were plotted inside a unit circle, with each vector’s position corresponding to its correlation with the components (**Supplementary Figure 9**)**.**

Stronger associations are depicted by vectors extending further away from the origin. In total, 31 ASVs grouped into 18 different genera, 115 metabolite peaks and 58 putative bacterial functions correlated positively (correlation ≥ 0.7). The bacterial genera represented included *Amphiplicatus, Bdellovibrio, Dokdonia, Hellea, Jannaschia, Peridibacter* as well as several genera affiliated with the *Micavibrionaceae, Rhizobiaceae, Rhodobacteraceae* and *Sphingomonadaceae* families. At the phylum level, nearly 80 % of these bacteria were assigned to *Pseudomonadota* with the great majority belonging to the *Alphaproteobacteria* class (52 %). The phyla *Bdellovibrionota* (9 %); *Bacteroidota* (6 %) and *Planctomycetota* (6 %) were also found. As for the metabolic peaks, only a putative identification based on mass-to-charge ratio (*m/z*) and retention time (min) was carried out. Several peaks appeared to be identified as phenolic compounds (flavonoids), terpenoids and lipids. Finally, the putative positively correlated bacterial functions were essentially synthesis functions (amino acids, nucleotides, sugars, lipids, vitamins) as well as energy production. The biosynthesis of pigments, particularly flavin, was also noted.

## 4 Discussion

The surface microbiota of macroalgae, including green macroalgae, is increasingly studied within the framework of the holobiont concept (Wichard, 2023). However, to the best of our knowledge, the intricate relationships between the green macroalgae *Ulva* spp. and its epiphytic bacterial communities, as well as the environmental factors influencing these interactions, remain poorly understood. In this study, the surface microbiota and metabolome of a pool of macroalgae from the *Ulva* genus were monitored over an 18-month period. A succession of four *Ulva* species was also observed at the sampling site.

### 4.1 *Ulva* spp. harboured a specific surface microbiota

The macroalgal epibacterial community structure was compared to bacterial communities from rocky substrate biofilm and seawater to assess host-specificity. A clear distinction emerged between habitats, as observed for *Ulva australis* (Bare Island, Australia) surface microbiota versus seawater planktonic bacteria (Burke et al.*,* 2011) and for *Ulva linza* surface microbiota versus rocky biofilm (Guo *et al*., 2022). Over 1.5 years, seawater bacterial planktonic communities showed less dispersion than those on algal surfaces and rocky biofilms. Algal epibacterial communities were closer to rocky biofilms, likely due to shared biofilm-forming bacteria, distinct from planktonic bacteria (Flemming et al.*,* 2016; Alsufyani et al.*,* 2017; Li et al.*,* 2019). Despite proximity between bacterial composition with predominance of *Alphaproteobacteria, Bacteroidia* and *Gammaproteobacteria,* differences were observed at lower taxonomic levels. The algal surface microbiota was dominated by unknown *Rhodobacteracea* and *Saprospiraceae,* as already described in *Ulva australis* (Burke et al., 2011). Other genera described in this study (*Granolusicoccus, Littorimonas, Hellea* and *Rudibimonas*) were also present in *Ulva lactuca* surface (Comba González et al., 2021). Seawater bacterial community was dominated by *Planktomarina* and *Amylibacter*, previously identified as part of the seawater core taxa community (Ul-Hasan et al., 2019; Paix et al., 2020; Chen et al., 2022). Rocky biofilms, in contrast, lacked a dominant genus (Pielou index ≈ 1) and had greater alpha-diversity as previously described in Guo *et al*., 2022. Both algal and rocky surfaces faced similar environmental pressures but showed distinct taxa due to active host selection. This could result from algal exudates like DMSP, which inhibits *Cytophaga* spp. fouling (Saha *et al*., 2012; Ingle *et al*., 2022) or acts as a chemotactic signal for *Roseovarius* sp. MS2, promoting *Ulva* development (Kessler et al.*,* 2018). Structural compounds like sulfated xyloarabinogalactans and ulvanes in algae (Abdul Malik et al., 2020) may also affect host selection and community specificity.

These patterns highlight host-specificity in the structure of *Ulva*-associated surface microbiota. However, it is important to acknowledge that our sampling included multiple *Ulva* species, which could not be distinguished in the field due to their morphological similarity. While this reflects the natural diversity of *Ulva* populations in situ*,* the temporal succession of *Ulva* species—previously documented, particularly in the context of green tides (Bermejo et al., 2019Bermejo *et*; Steinhagen et al.*,* 2019) introduces a degree of taxonomic heterogeneity that may influence microbiota composition and adds complexity to the interpretation of host-specific microbial patterns.

### 4.2 Demonstration of a constant bacterial community associated with the algal surface

On all sampling dates and stations, 24 genera, representing at least 50 % of total relative bacterial abundance, were consistently detected. This led to the investigation of a constant bacterial community over a 1.5-year period. Taxonomic similarities were noted with other studies on *Ulva* spp.associated bacteria. Comba González et al. (2021) identified a consistent core microbiota on *Ulva lactuca* over two years using 16S rRNA sequencing. They mentioned several taxonomic affiliations in common (order level) with the present study (*Flavobacteriales, Caulobacterales, Chromatiales, Cytophagales* and *Rhodobacterales*). At the genus level, Van der Loos *et al*. (2023) found a small core microbiota, including *Sulfitobacter* and uncultured *Rhodobacteraceae*, across 15 distinct *Ulva* taxa along 2000 km of Baltic coastline. More recently, Liu et al. (2023) identified a year-round core microbiota in *Ulva* spp. with 14 genera, half of which overlap with those in this study (e.g., *Algitalea, Hellea, Lewinella, Sulfitobacter, Rhodobacteraceae* unc*., Saprospiraceae* unc., *Microtrichaceae* unc.), including *Maribacter, Rubidimonas,* and *Yoonia-Loktanella* as dominant taxa. Other known *Ulva* epiphytic bacteria found in this study's core microbiota include *Algitalea* (Yoon et al.*,* 2015), *Hellea* (Tujula et al.*,* 2010; Guo et al.*,* 2022), *Litorimonas,* and *Dokdonia,* associated with high-salinity *Ulva* environments (Van der Loos *et al*., 2023).The influence of specific environmental factors (salinity, temperature) on the bacterial communities associated with *Ulva* has already been proven, and a consensus is emerging on the dynamic nature of the composition of the *Ulva* microbiota, from both a taxonomic and a functional point of view (Ghaderiardakani *et al*., 2020; Van der Loos et al.*,* 2023; Wichard, 2023). However, the existence of a taxonomic core microbiota remains debated, with studies supporting (Lachnit et al., 2009; Tujula et al., 2010, 2011; Comba González et al., 2021) or rejecting (e.g., Burke *et al*., 2011) this concept. Several studies have demonstrated the presence of macroalgae-specific bacterial communities, along with temporally adapted epiphytic bacterial abundances over time (Lachnit *et al*., 2009, 2011; Comba González et al., 2021; Guo et al.*,* 2022). These findings suggest a functional role for these bacterial communities in association with macroalgae. Strains belonging to the genera *Maribacter* (*Maribacter* sp. MS6) and *Roseovarius* (*Roseovarius* sp. MS2)*,* have been identified as essential to the morphogenesis of *Ulva mutabilis*, forming a tripartite community with the alga (Spoerner et al., 2012; Grueneberg et al., 2016; Ghaderiardakani et al., 2017). Other key *Ulva* functions (e.g., reproduction, defense) depend on genera such as *Maribacter, Sulfitobacter*, and *Polaribacter* (Manley and Barbero, 2001; Joint et al.*,* 2002), found in this study's core microbiota. *Maribacter* produces the morphogenesis factor thallusin (Matsuo et al.*,* 2003); *Sulfitobacter* supports *Ulva mutabilis* growth (Grueneberg *et al*., 2016*); Polaribacter* promotes *Ulva fenestrata* growth (Nedashkovskaya *et al*., 2013). Chemical signaling between the host and microorganisms reveals the intricate specificity of the microbiota-host relationship and underscores the functional role of the microbiota. Host-microbiota interactions involve chemical molecules like N-acylhomoserine lactones (AHLs), biofilm communication molecules that attract *Ulva* zoospores; however quorum sensing disruption through autoinducer inactivation (AiiA) proteins can nullify this effect (Tait *et al*., 2005; Joint et al.*,* 2007). The hypothesis of a core microbiota with a global functional role (Burke et al.*,* 2011) may also be supported by the consistently high abundance (59 %−92.8 %) of the constant bacterial community associated with *Ulva* spp. in this study. The 24 genera in the core microbiota include 1491 ASVs, representing 30 % of total ASVs but only 4 % of genera identified. Recent studies have applied ecological models from plants and animals to host-associated microbial communities (Roth-Schulze et al.*,* 2016). Burke et al. (2011) found high taxonomic variability in *Ulva australis* epibiotic communities but strong functional similarities. Roth-Schulze et al. (2018) later identified a shared gene pool across *Ulva* species and biogeographies, potentially reflecting random colonization by a "functional guild." This aligns with the competitive lottery model (Sale, 1978), suggesting core microbiota genera perform essential functions for algae. Changes in bacterial abundance, both core and variable, likely aid *Ulva*'s environmental adaptability.

### 4.3 Environmental parameters and host physiology as drivers to explain variations of *Ulva* spp. epiphytic bacteria

This study identified salinity, nitrate concentration, and precipitation as the main environmental drivers influencing microbial community shifts. Seasonal clustering of bacterial communities was observed, with distinct compositions in spring, summer, fall, and winter. These dynamics are consistent with previous findings for brown (Michelou et al., 2013; Paix et al., 2019), red (Lachnit et al., 2011; Lu et al., 2023), and green macroalgae (Comba González et al., 2021; Lu et al.*,* 2023). For instance, Lachnit *et* al. (2011) reported recurring bacterial patterns in *Ulva intestinalis* between summer and winter, while Comba González et al. (2021) linked changes in *Ulva lactuca* microbiota to rising seawater temperatures. A similar pattern was observed in a three-years study on *Gracilaria vermiculophylla* where the epibiotic microbiota exhibited predictable, seasonally driven taxonomic and functional shifts, with a seasonal core microbiota reappearing at specific times and a permanent core persisting year-round regardless of season or location (Mudlaff et al.*,* 2025).

In our study, temperature-related patterns were also evident. A higher abundance of *Paraglaciecola*—a genus common in cold waters (Luhtanen et al.*,* 2018)was observed in February (8.4 °C), reinforcing the role of seawater temperature. Similarly, salinity influenced bacterial composition: low-salinity samples were dominated by *Luteolibacter, Cyanobium, Pirellula*, and *Lacihabitans*, whereas high-salinity samples were enriched in *Litorimonas, Leucothrix, Sulfurovum, Algibacter*, and *Dokdonia* (Van der Loos et al.*,* 2023). The microbiota in our study most closely resembled the high-salinity assemblage.

Seasonal changes also affected the abundance of both core and rare taxa, likely reflecting bacterial adaptations to changing environmental conditions. Cold-adapted genera such as *Paraglaciecola* and *Psychromonas*, typically found in polar or deep-sea environments (Lauro et al.*,* 2013; Bech et al.*,* 2017), peaked in February 2021. *Paraglaciecola* is also known to degrade ulvan from *Ulva* surfaces (Tanaka *et al*., 2022). The denitrifier *Truepera* showed high relative abundance during nitrate peaks in winter (Feb. 2021/2022, Dec. 2021), while *Sphingorhabdus*—a UV-resistant genus (Coppola et al.*,* 2023) peaked in summer, especially June 2022, likely in response to elevated temperature and solar radiation. Bacterial communities likely aid macroalgae in adapting to environmental stresses.

This study also explored the role of *Ulva* spp. physiology, including neutral sugars, uronic acids, lipids, proteins, and pigments, in shaping microbial communities. Variations in uronic acid content, lipid levels, and algal humidity appeared to be correlated with shifts in bacterial community structure. Uronic acids, a major cell wall component, were higher in summer and significantly correlated with bacterial responses (*p*< 0.05, R² = 56 %). An increase in uronic acids in summer could mean that the cell wall of *Ulva* spp. is thicker, which would be an advantage for bacteria with the enzymatic machinery to use them. According to the literature, *Flavobacterium* and *Alteromonas* genera (both represented in *Ulva* spp. microbiota in this study) can use uronic acids in their metabolism (Karapally and Dietrich, 1970; Coste et al., 2015; Inoue et al., 2015). However, we acknowledge that this correlation does not imply a direct causal relationship. It remains unclear whether these physiological traits shape the microbial community or whether specific bacterial groups may themselves influence host physiology—for instance by degrading uronic acids or modulating surface properties.

### 4.4 Evidence of seasonal co-variation between *Ulva* spp. epiphytic bacteria and surface metabolome

The surface metabolome of *Ulva* spp. showed a clustering pattern aligned with the seasonal behavior of algal epiphytic bacteria. Seasonality in antifouling/antibacterial activities has been previously observed in green macroalgae extracts, particularly in *Ulva rigida*, where antibacterial and antioxidant activities peak in spring and summer due to higher temperature and fouling pressure (Hellio et al.*,* 2004; Trigui *et al*., 2013). Non-targeted metabolomics has enhanced the understanding of the seasonal variations in the chemical composition of algae**.** For instance, monthly variations of the metabolome over a 13-month survey were demonstrated in 4 species of the brown macroalgae *Lobophora*, these variations being positively correlated with sea surface temperature and salinity (Gaubert et al., 2019). Chemical surface landscape in brown macroalgae *Fucus* also exhibits seasonal differences between spring/summer and autumn/winter, months when temperature and light are the main environmental drivers (Rickert et al., 2016). In the current study, factors such as irradiation time, solar radiation, chlorophyll-a, temperature and salinity were identified as key drivers of *Ulva* spp. surface metabolome. These were correlated with several metabolites, including potential chlorine molecules. In the current study, an integrative analysis of the surface metabolome, microbiota, and predictive bacterial function datasets revealed a positive correlation (correlation ≥ 0.7) between 31 amplicon sequence variants (ASVs) classified into 18 genera, 115 metabolite peaks, and 58 bacterial functions. These findings suggest that over a period of one and a half years of sampling, certain bacterial taxa exhibited strong associations with the presence of specific chemical compounds on the surface of *Ulva* spp., further supporting the connection previously established between epiphytic bacteria and the surface metabolome.

June 2022, a distinct chemical profile emerged, with increased levels of DMSP and proline-betaine, which are known to function as protective molecules against stresses (salinity, oxidative, temperature) in macroalgae (Sunda et al.*,* 2002; Carrión *et al*., 2023). Both DMSP and proline-betaine have previously been identified as discriminative metabolites in the seasonal monitoring of the surface metabolome of the brown alga *Taonia atomaria* (Paix et al.*,* 2019). These compounds are notably elevated during the summer months in brown macroalgae (Paix *et al*., 2019; Carvalho *et al*., 2022). Seasonal shifts in bacterial communities were also observed, with *Dokdonia* reaching its peak abundance in June 2022, coinciding with high solar radiation levels (Pearson’s correlation coefficient = 0.56; *p*≤ 0.05) and the presence of antioxidant metabolites. *Dokdonia* is representative of the *Flavobacteriia* class and some of them contain proteorhodopsin (PRs), which are membrane proteins with retinal protein as light absorbing molecules (González et al., 2011). Those bacterial light-dependent proton pumps allow bacteria to use light as a source of energy (photoheterotrophy) for maintenance as well as active cell growth (Gómez-Consarnau *et al*., 2007). This correlation supports the hypothesis that solar radiation influences the surface microbiota and is reinforced by the presence of peroxiredoxins in bacterial functions which protect against cellular oxidative damages (Dubbs and Mongkolsuk, 2007). Another notable example of the link between microbiota dynamics and the surface metabolome was observed in April 2021 and April 2022, as both months exhibited similar surface microbiota compositions and metabolome profiles. Indeed, the core microbiota accounted for 92.8 %, with *Rhodobacteraceae* representing 35.3 % of total relative abundance in April 2021 and 30.7 % in April 2022. *Rhodobacteraceae*, known for their adaptation to an epiphytic lifestyle (Dogs et al.*,* 2017) and temporal shifts in abundance (Comba González et al., 2021), contain members with a biosynthetic pathway for vitamin B12 (Sañudo-Wilhelmy et al.*,* 2014), which may influence algal growth (Fries, 1993; Dogs et al.*,* 2017). The peak abundance of *Rhodobacteraceae* coincided with *Ulva* growth in March/April, as observed in previous studies (Rosenberg and Ramus, 1982; Coffaro and Sfriso, 1997). These findings highlight the potential link between microbiota and host physiological needs, emphasizing metabolic complementarity.

In conclusion, the multi-omics approach is particularly relevant for studying holobiont dynamics, suggesting that surface-associated bacteria of *Ulva* spp. are distinct from those in the surrounding environment. Changes in the relative abundances of the bacterial community were observed, affecting both the core microbiota and rare taxa. Environmental factors, as well as host physiology, were identified as potential drivers that partially explain the seasonal dynamics of *Ulva* spp.associated bacteria. Seasonal variations were also detected in the surface metabolome, with covariations observed between surface bacteria and the metabolome, indicating potential responses to environmental stresses. A deeper understanding of the coupled dynamics of surface microbiota and metabolome over time will enhance our knowledge of the functioning of the macroalgal holobiont in response to environmental variations, which is particularly relevant in the context of climate change.

## Author contributions

**Sauvann Paulino**: conceptualization, formal analysis, investigation, methodology, visualization, writing – original draft. **Cyril Noel**: formal analysis, methodology, visualization. **Laura Rieusset:** formal analysis, visualization. **Laure Taupin**: formal analysis, investigation. **Gwenaelle Le Blay:** conceptualization, funding acquisition, supervision, writing – original draft. **Nathalie Bourgougnon:** conceptualization, funding acquisition, supervision, writing – original draft.

## Declaration of competing interest

The authors declare that they have no known competing financial interests or personal relationships that could have appeared to influence the work reported in this paper.

## Data Availability

Sequences data were deposited and are publicly available in the European Nucleotide Archive (ENA) under the BioProject PRJEB85979, accession number. https://www.ebi.ac.uk/ena/browser/view/PRJEB85979

## Appendix Supplementary materials

Image, application 1
