# Supplementary material for: Drivers of seasonal dynamics in Ulva spp. associated microbiota and surface metabolome: The interplay between environment and host physiology
Source: Curr Res Microb Sci. 2025 Jul 11;9:100439. doi: 10.1016/j.crmicr.2025.100439 (PMC12304696; doi:10.1016/j.crmicr.2025.100439)
Supplement: Supplementary file 1 [file mmc1.docx]

**Supplementary information**

Drivers of seasonal dynamics in *Ulva* spp.-associated microbiota and surface metabolome.: the interplay between environment and host physiology

Sauvann Paulino ^1, 2^, Cyril Noël ^3^, Laura Rieusset ^1^, Laure Taupin ^1^, Gwenaëlle Le Blay ^2^, Nathalie Bourgougnon ^1^

(1) UBS, Laboratoire de Biotechnologie et Chimie Marines, EMR CNRS 6076, Vannes/Lorient, France

(2) Univ Brest, CNRS, IRD, Ifremer, LEMAR, F-29290, Plouzané, France

(3) IFREMER-IRSI-Service de Bioinformatique (SeBiMER), Plouzané, France

Corresponding author at : UMR-CNRS 6539- Laboratoire des Sciences de l’Environnement Marin (LEMAR), Institut Universitaire et Europeen de la Mer (IUEM), Rue Dumont d’Urville, 29280 Plouzane, France. Tel: +33 02 90 91 55 65 ; E-mail: gwenaelle.leblay@univ-brest.fr

## **
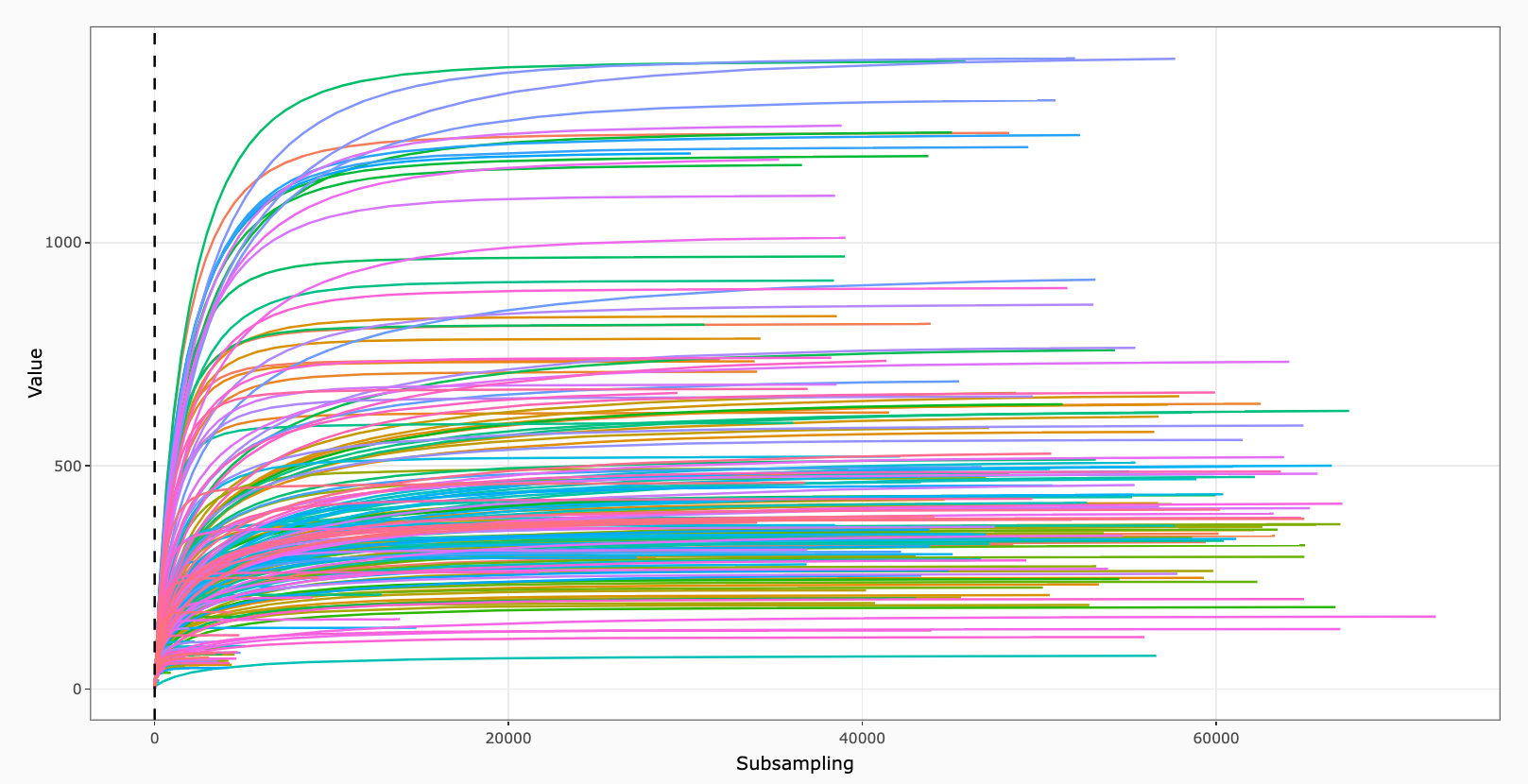
Supplementary information about the 16S amplicon sequencing**

Supplementary Figure 1 : Rarefaction curves obtained after data processing of 16S rRNA gene sequences

## **Supplementary information for characterization of *Ulva***

Specimens of algae collected between February 2021 and February 2022 were identified using molecular tools. **Supplementary Figure 2** shows the species affiliated with the genus *Ulva* found during this period at the sampling stations on Kerleven beach.


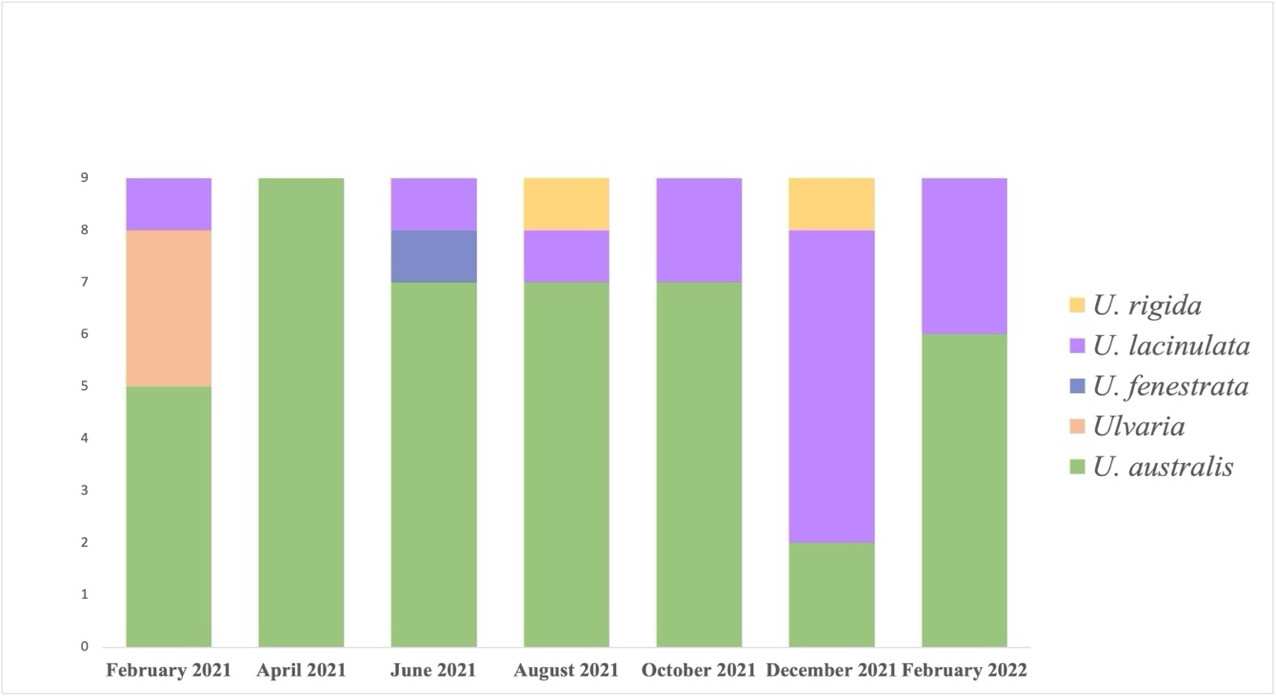


Supplementary Figure 2 : Respective numbers of the 4 species associated with the Ulva and Ulvaria genera from February 2021 to February 2022

Supplementary Table 1 : Identification of macroalgal specimens collected in the field between February 2021 and February 2022 using *tufA* gene sequencing

| Samples | Identification | Query cover | Identity (%) | Accession | Sequence length (bp) |
| --- | --- | --- | --- | --- | --- |
| Fev21-S1-U1 | *Ulva australis* | 100% | 100.00% | [MT160640.1](https://www.ncbi.nlm.nih.gov/nucleotide/MT160640.1?report=genbank&log$=nucltop&blast_rank=1&RID=3B0DJX10013) | 882 |
| Fev21-S1-U2 | *Ulva lacinulata* | 100% | 100.00% | [NC_053614.1](https://www.ncbi.nlm.nih.gov/nucleotide/NC_053614.1?report=genbank&log$=nucltop&blast_rank=1&RID=3B0KCC22016) | 883 |
| Fev21-S1-U3 | *Ulva australis* | 100% | 100.00% | [MT160640.1](https://www.ncbi.nlm.nih.gov/nucleotide/MT160640.1?report=genbank&log$=nucltop&blast_rank=1&RID=3B0P4MXA013) | 868 |
| Fev21-S2-U1 | *Ulvaria* sp. | / | 100.00% | / | 768 |
| Fev21-S2-U2 | *Ulvaria* sp. | / | 100.00% | / | 771 |
| Fev21-S2-U3 | *Ulvaria* sp. | / | 100.00% | / | 887 |
| Fev21-S3-U1 | *Ulva australis* | 100% | 100.00% | [MT160640.1](https://www.ncbi.nlm.nih.gov/nucleotide/MT160640.1?report=genbank&log$=nucltop&blast_rank=1&RID=3B19TG9E016) | 889 |
| Fev21-S3-U2 | *Ulva australis* | 100% | 100.00% | [MT160640.1](https://www.ncbi.nlm.nih.gov/nucleotide/MT160640.1?report=genbank&log$=nucltop&blast_rank=1&RID=3B19TG9E016) | 900 |
| Fev21-S3-U3 | *Ulva australis* | 99.78% | 100.00% | [MT160640.1](https://www.ncbi.nlm.nih.gov/nucleotide/MT160640.1?report=genbank&log$=nucltop&blast_rank=1&RID=3B1CXA1P016) | 843 |
| Avr21-S1-U1 | *Ulva australis* | 99.88% | 100.00% | [MT160640.1](https://www.ncbi.nlm.nih.gov/nucleotide/MT160640.1?report=genbank&log$=nucltop&blast_rank=1&RID=3B1F0DK2013) | 903 |
| Avr21-S1-U2 | *Ulva australis* | 99.67% | 100.00% | [MT160640.1](https://www.ncbi.nlm.nih.gov/nucleotide/MT160640.1?report=genbank&log$=nucltop&blast_rank=1&RID=3B1F0DK2013) | 885 |
| Avr21-S1-U3 | *Ulva australis* | 99.77% | 100.00% | [MT160640.1](https://www.ncbi.nlm.nih.gov/nucleotide/MT160640.1?report=genbank&log$=nucltop&blast_rank=1&RID=3B1F0DK2013) | 760 |
| Avr21-S2-U1 | *Ulva australis* | / | / | / | / |
| Avr21-S2-U2 | *Ulva australis* | 100% | 99.87% | [MT160640.1](https://www.ncbi.nlm.nih.gov/nucleotide/MT160640.1?report=genbank&log$=nucltop&blast_rank=1&RID=3B1YGGTX013) | 890 |
| Avr21-S2-U3 | *Ulva australis* | 100% | 99.78% | [MT160640.1](https://www.ncbi.nlm.nih.gov/nucleotide/MT160640.1?report=genbank&log$=nucltop&blast_rank=1&RID=3B224Z76013) | 902 |
| Avr21-S3-U1 | *Ulva australis* | 100% | 99.67% | [MT160640.1](https://www.ncbi.nlm.nih.gov/nucleotide/MT160640.1?report=genbank&log$=nucltop&blast_rank=1&RID=3B26S2Z5016) | 762 |
| Avr21-S3-U2 | *Ulva australis* | 100% | 99.87% | [MT160640.1](https://www.ncbi.nlm.nih.gov/nucleotide/MT160640.1?report=genbank&log$=nucltop&blast_rank=1&RID=3B26S2Z5016) | 676 |
| Avr21-S3-U3 | *Ulva australis* | 100% | 99.85% | [MT160640.1](https://www.ncbi.nlm.nih.gov/nucleotide/MT160640.1?report=genbank&log$=nucltop&blast_rank=1&RID=3B26S2Z5016) | 901 |
| Jun21-S1-U1 | *Ulva australis* | 100% | 99.56% | [MT160640.1](https://www.ncbi.nlm.nih.gov/nucleotide/MT160640.1?report=genbank&log$=nucltop&blast_rank=1&RID=3B26S2Z5016) | 893 |
| Jun21-S1-U2 | *Ulva fenestrata* | 100% | 99.89% | [MT160662.1](https://www.ncbi.nlm.nih.gov/nucleotide/MT160662.1?report=genbank&log$=nucltop&blast_rank=1&RID=3B1PD6CB016) | 883 |
| Jun21-S1-U3 | *Ulva australis* | 100% | 99.89% | [MT160640.1](https://www.ncbi.nlm.nih.gov/nucleotide/MT160640.1?report=genbank&log$=nucltop&blast_rank=1&RID=3B26S2Z5016) | 675 |
| Jun21-S2-U1 | *Ulva australis* | 100% | 99.87% | [MT160640.1](https://www.ncbi.nlm.nih.gov/nucleotide/MT160640.1?report=genbank&log$=nucltop&blast_rank=1&RID=3B26S2Z5016) | 753 |
| Jun21-S2-U2 | *Ulva australis* | 100% | 100.00% | [MT160640.1](https://www.ncbi.nlm.nih.gov/nucleotide/MT160640.1?report=genbank&log$=nucltop&blast_rank=1&RID=3B26S2Z5016) | 876 |
| Jun21-S2-U3 | *Ulva australis* | 100% | 100.00% | [MT160640.1](https://www.ncbi.nlm.nih.gov/nucleotide/MT160640.1?report=genbank&log$=nucltop&blast_rank=1&RID=3B26S2Z5016) | 735 |
| Jun21-S3-U1 | *Ulva australis* | 100% | 99.85% | [MT160640.1](https://www.ncbi.nlm.nih.gov/nucleotide/MT160640.1?report=genbank&log$=nucltop&blast_rank=1&RID=3B26S2Z5016) | 677 |
| Jun21-S3-U2 | *Ulva australis* | / | / | / | / |
| Jun21-S3-U3 | *Ulva lacinulata* | 100% | 99.89% | [NC_053614.1](https://www.ncbi.nlm.nih.gov/nucleotide/NC_053614.1?report=genbank&log$=nucltop&blast_rank=1&RID=3B3C2XAM013) | 1560 |
| Aou21-S1-U1 | *Ulva australis* | 100% | 100.00% | [MT160640.1](https://www.ncbi.nlm.nih.gov/nucleotide/MT160640.1?report=genbank&log$=nucltop&blast_rank=1&RID=3B26S2Z5016) | 668 |
| Aou21-S1-U2 | *Ulva lacinulata* | 100% | 99.17% | [MT160657.1](https://www.ncbi.nlm.nih.gov/nucleotide/MT160657.1?report=genbank&log$=nucltop&blast_rank=3&RID=3BSVCGBB013) | 841 |
| Aou21-S1-U3 | *Ulva australis* | / | / | / | / |
| Aou21-S2-U1 | *Ulva australis* | 100% | 99.85% | [MT160640.1](https://www.ncbi.nlm.nih.gov/nucleotide/MT160640.1?report=genbank&log$=nucltop&blast_rank=1&RID=3B26S2Z5016) | 679 |
| Aou21-S2-U2 | *Ulva australis* | 100% | 99.89% | [MT160640.1](https://www.ncbi.nlm.nih.gov/nucleotide/MT160640.1?report=genbank&log$=nucltop&blast_rank=1&RID=3B26S2Z5016) | 902 |
| Aou21-S2-U3 | *Ulva australis* | 100% | 99.89% | [MT160640.1](https://www.ncbi.nlm.nih.gov/nucleotide/MT160640.1?report=genbank&log$=nucltop&blast_rank=1&RID=3B26S2Z5016) | 894 |
| Aou21-S3-U1 | *Ulva australis* | 100% | 99.88% | [MT160640.1](https://www.ncbi.nlm.nih.gov/nucleotide/MT160640.1?report=genbank&log$=nucltop&blast_rank=1&RID=3B26S2Z5016) | 834 |
| Aou21-S3-U2 | *Ulva rigida* | 100% | 100% | [MT160670.1](https://www.ncbi.nlm.nih.gov/nucleotide/MT160670.1?report=genbank&log$=nucltop&blast_rank=1&RID=3C8H0CBH016) | 783 |
| Aou21-S3-U3 | *Ulva australis* | 100% | 99.87% | [MT160640.1](https://www.ncbi.nlm.nih.gov/nucleotide/MT160640.1?report=genbank&log$=nucltop&blast_rank=1&RID=3B26S2Z5016) | 785 |
| Oct21-S1-U1 | *Ulva australis* | 100% | 99.43% | [MT160640.1](https://www.ncbi.nlm.nih.gov/nucleotide/MT160640.1?report=genbank&log$=nucltop&blast_rank=1&RID=3B26S2Z5016) | 699 |
| Oct21-S1-U2 | *Ulva lacinulata* | 100% | 99.67% | [MT160657.1](https://www.ncbi.nlm.nih.gov/nucleotide/MT160657.1?report=genbank&log$=nucltop&blast_rank=5&RID=3C9ADMFW016) | 903 |
| Oct21-S1-U3 | *Ulva australis* | 100% | 99.67% | [MT160640.1](https://www.ncbi.nlm.nih.gov/nucleotide/MT160640.1?report=genbank&log$=nucltop&blast_rank=1&RID=3C9C5G67013) | 903 |
| Oct21-S2-U1 | *Ulva australis* | 100% | 100% | [MT160640.1](https://www.ncbi.nlm.nih.gov/nucleotide/MT160640.1?report=genbank&log$=nucltop&blast_rank=1&RID=3C9C5G67013) | 884 |
| Oct21-S2-U2 | *Ulva australis* | / | / | / | / |
| Oct21-S2-U3 | *Ulva australis* | 100% | 99.55% | [MT160640.1](https://www.ncbi.nlm.nih.gov/nucleotide/MT160640.1?report=genbank&log$=nucltop&blast_rank=1&RID=3C9C5G67013) | 654 |
| Oct21-S3-U1 | *Ulva australis* | 100% | 99.85% | [MT160640.1](https://www.ncbi.nlm.nih.gov/nucleotide/MT160640.1?report=genbank&log$=nucltop&blast_rank=1&RID=3C9C5G67013) | 895 |
| Oct21-S3-U2 | *Ulva lacinulata* | 100% | 99.78% | [NC_053614.1](https://www.ncbi.nlm.nih.gov/nucleotide/NC_053614.1?report=genbank&log$=nucltop&blast_rank=1&RID=3CA39EUG016) | 900 |
| Oct21-S3-U3 | *Ulva australis* | 100% | 99.78% | [MT160640.1](https://www.ncbi.nlm.nih.gov/nucleotide/MT160640.1?report=genbank&log$=nucltop&blast_rank=1&RID=3C9C5G67013) | 902 |
| Dec21-S1-U1 | *Ulva lacinulata* | 100% | 99.67% | [NC_053614.1](https://www.ncbi.nlm.nih.gov/nucleotide/NC_053614.1?report=genbank&log$=nucltop&blast_rank=1&RID=3CAA6P3T016) | 900 |
| Dec21-S1-U2 | *Ulva lacinulata* | 100% | 99.78% | [NC_053614.1](https://www.ncbi.nlm.nih.gov/nucleotide/NC_053614.1?report=genbank&log$=nucltop&blast_rank=1&RID=3CACAJW6016) | 899 |
| Dec21-S1-U3 | *Ulva lacinulata* | 100% | 100% | [NC_053614.1](https://www.ncbi.nlm.nih.gov/nucleotide/NC_053614.1?report=genbank&log$=nucltop&blast_rank=1&RID=3CAKCPR2013) | 899 |
| Dec21-S2-U1 | *Ulva rigida* | 100% | 99.78% | [MT160670.1](https://www.ncbi.nlm.nih.gov/nucleotide/MT160670.1?report=genbank&log$=nucltop&blast_rank=1&RID=3CARK7JB016) | 888 |
| Dec21-S2-U2 | *Ulva australis* | 100% | 99.66% | [MT160640.1](https://www.ncbi.nlm.nih.gov/nucleotide/MT160640.1?report=genbank&log$=nucltop&blast_rank=1&RID=3CB5E79D013) | 898 |
| Dec21-S2-U3 | *Ulva australis* | 100% | 99.89% | [MT160640.1](https://www.ncbi.nlm.nih.gov/nucleotide/MT160640.1?report=genbank&log$=nucltop&blast_rank=1&RID=3CB79JGD016) | 896 |
| Dec21-S3-U1 | *Ulva lacinulata* | 100% | 99.89% | [NC_053614.1](https://www.ncbi.nlm.nih.gov/nucleotide/NC_053614.1?report=genbank&log$=nucltop&blast_rank=1&RID=3CB97921016) | 898 |
| Dec21-S3-U2 | *Ulva lacinulata* | 100% | 99.89% | [NC_053614.1](https://www.ncbi.nlm.nih.gov/nucleotide/NC_053614.1?report=genbank&log$=nucltop&blast_rank=1&RID=3CBBPPHA016) | 903 |
| Dec21-S3-U3 | *Ulva lacinulata* | 100% | 99.78% | [NC_053614.1](https://www.ncbi.nlm.nih.gov/nucleotide/NC_053614.1?report=genbank&log$=nucltop&blast_rank=1&RID=3CBDGN4J016) | 675 |
| Fev22-S1-U1 | *Ulva australis* | 100% | 100% | [MT160640.1](https://www.ncbi.nlm.nih.gov/nucleotide/MT160640.1?report=genbank&log$=nucltop&blast_rank=1&RID=3CBFC3X6016) | 619 |
| Fev22-S1-U2 | *Ulva australis* | 100% | 100% | [KF195528.1](https://www.ncbi.nlm.nih.gov/nucleotide/KF195528.1?report=genbank&log$=nucltop&blast_rank=1&RID=3CBGT3AK013) | 885 |
| Fev22-S1-U3 | *Ulva lacinulata* | 100% | 100% | [NC_053614.1](https://www.ncbi.nlm.nih.gov/nucleotide/NC_053614.1?report=genbank&log$=nucltop&blast_rank=1&RID=3CBSVNP0016) | 886 |
| Fev22-S2-U1 | *Ulva australis* | 100% | 100% | [MT160640.1](https://www.ncbi.nlm.nih.gov/nucleotide/MT160640.1?report=genbank&log$=nucltop&blast_rank=1&RID=3CBU8NUC016) | 673 |
| Fev22-S2-U2 | *Ulva australis* | 100% | 100% | [MT160640.1](https://www.ncbi.nlm.nih.gov/nucleotide/MT160640.1?report=genbank&log$=nucltop&blast_rank=1&RID=3CBXSJYU016) | 886 |
| Fev22-S2-U3 | *Ulva australis* | 100% | 100% | [MT160640.1](https://www.ncbi.nlm.nih.gov/nucleotide/MT160640.1?report=genbank&log$=nucltop&blast_rank=1&RID=3CBZBN7X013) | 601 |
| Fev22-S3-U1 | *Ulva lacinulata* | 100% | 100% | [MT160657.1](https://www.ncbi.nlm.nih.gov/nucleotide/MT160657.1?report=genbank&log$=nucltop&blast_rank=1&RID=3CC124C6016) | 613 |
| Fev22-S3-U2 | *Ulva lacinulata* | 100% | 100% | [MT895098.1](https://www.ncbi.nlm.nih.gov/nucleotide/MT895098.1?report=genbank&log$=nucltop&blast_rank=1&RID=3CC2JGH5016) | 749 |
| Fev22-S3-U3 | *Ulva australis* | / | / | / | / |


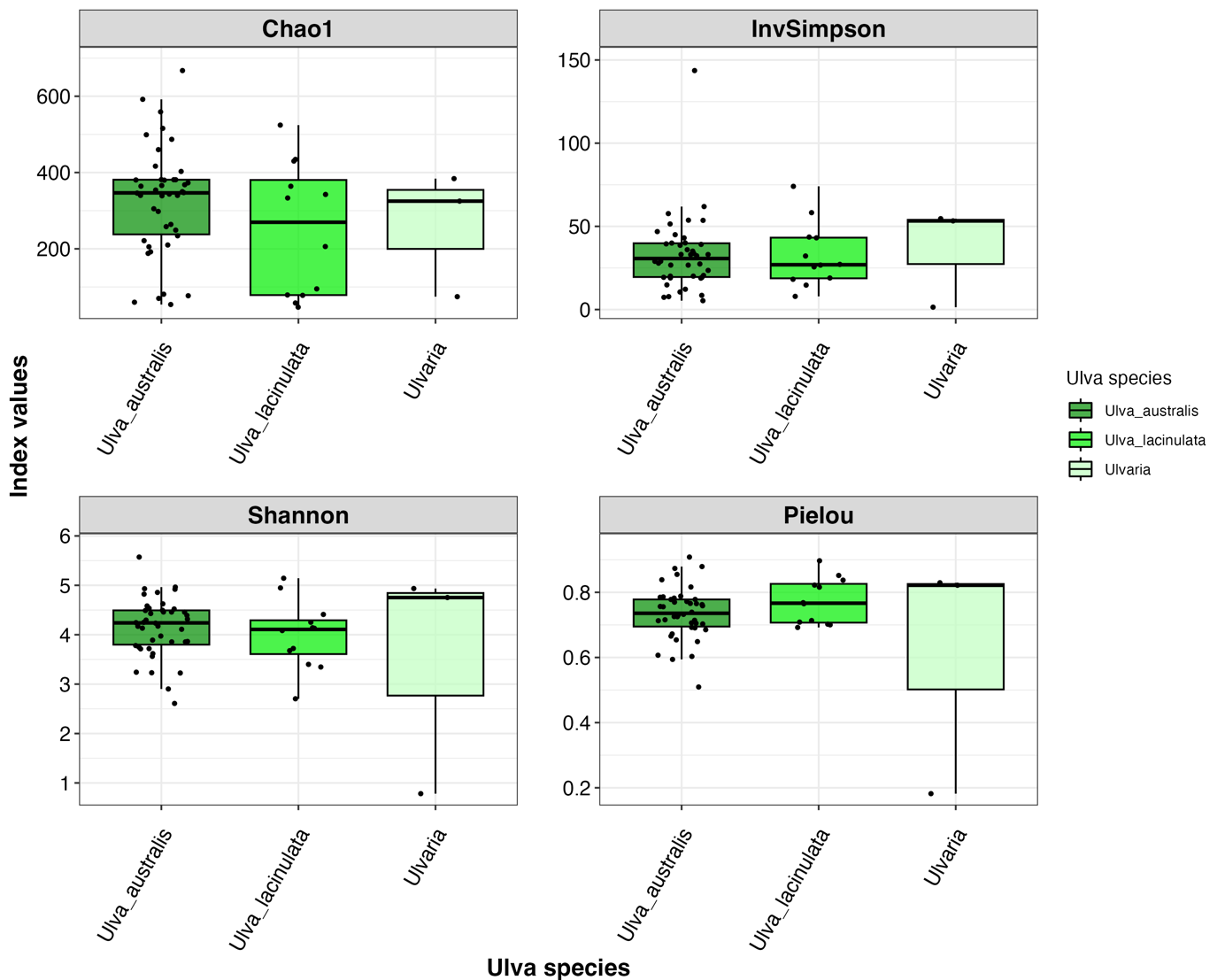


*Supplementary Figure 3 : Epibacterial alpha diversity measured using Chao1, Inverse Simpson, Shannon, and Pielou indices based on 16S metabarcoding data, according to the macroalgal species collected in the field. One-way ANOVA followed by post hoc HSD tests was used to assess significant differences among host species.*


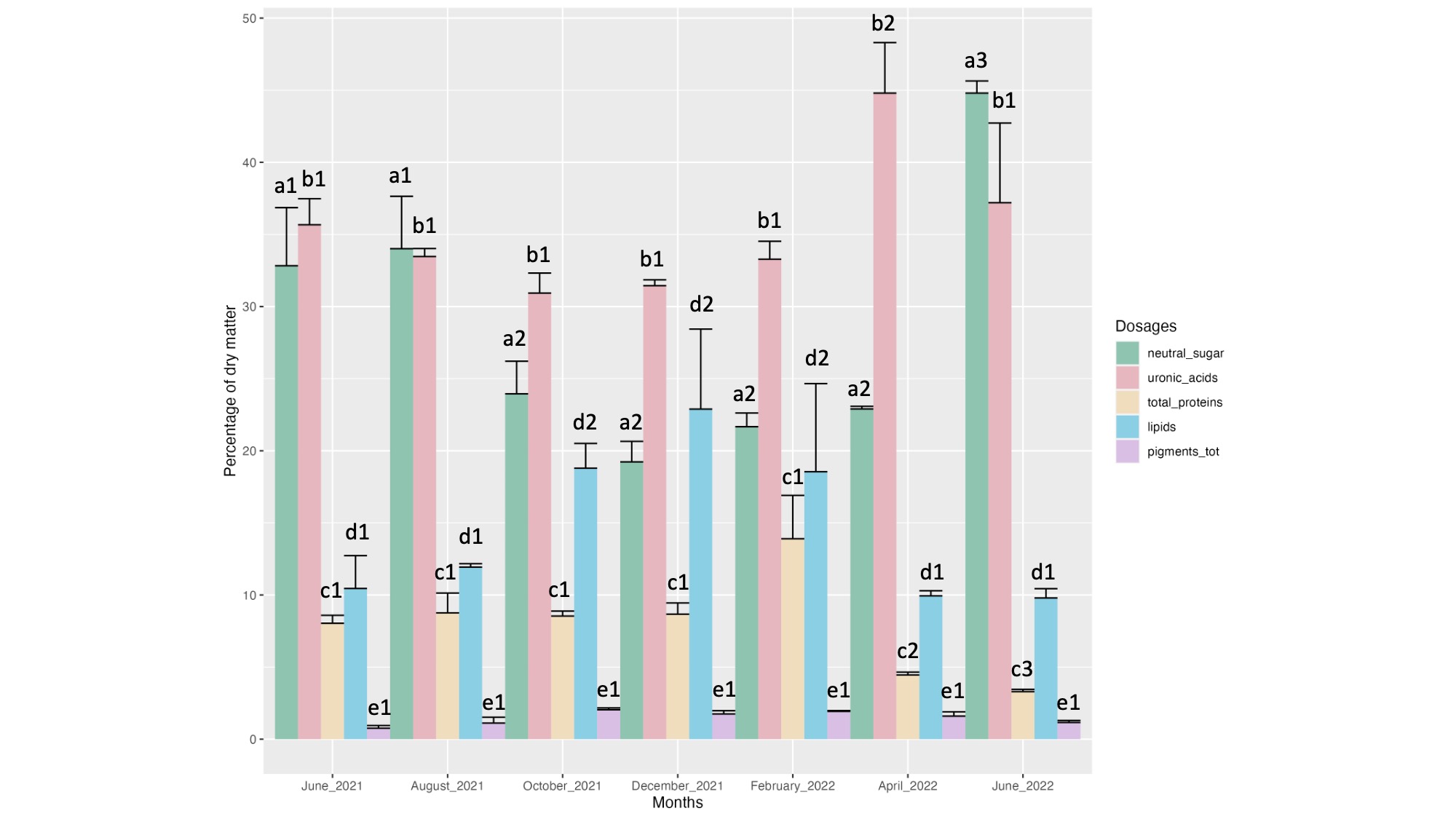


Supplementary Figure 4 : Biochemical composition analysis of neutral sugars (green), uronic acids (pink), total proteins (beige), lipids (blue) and total pigments (purple) between June 2021 and June 2022 in percentage of algal dry matter. Biochemical analysis was carried out in triplicate.

Moreover, biochemical analysis were conducted to characterize the raw matter of *Ulva* spp., 10 mg of freeze-dried matter of each sample were mixed with 5 mL HCl 1M in a sealed vial. The acid extraction was performed at 100°C for 2 h, after which 5 mL NaOH 1M were added. The final solution was used to measure the neutral sugars, uronic acids, and proteins contents. Neutral sugars were determined by the phenol sulphuric acid method described by Dubois *et al*. (1956). Uronic acids were determined by using the meta-hydroxy-di-phenyl (MHDP) method (Blumenkrantz et Asboe-Hansen, 1973). Lipids were extracted with a mixture of chloroform/methanol (1:1, v/v) over 2 days under agitation. The extract was filtered and washed with distilled water. The lipid content was determined gravimetrically by Bligh and Dyer method (1959), whereas total pigments (chlorophyll, carotenoids) analyzed were done following Schmid and Stengel (2015). All tests were carried out in three biological replicates. One-way Anova was used to compare the means of each parameters between the months followed by a *post hoc* test (Tukey’s Honest Significance Difference, HSD, test) to assess the significance of differences between pairs of group means. All the test were calculated using R software (R version 4.2.3).

Several parameters linked to the physiology of the algal host (neutral sugars, uronic acids, total proteins, lipids and pigments) were monitored from June 2021 to June 2022.

A significant difference in the general state of the algae was observed from one month to the next (anova, *p* < 0.05). As expected, lipids were significantly higher in winter (December/February 2021) and a significant decrease was observed in the total proteins content in April/June 2022 ; neutral sugars increased markedly in summer (June 2021 and 2022/August 2021), while uronic acids increased mainly in April 2022 (*post hoc* test, HSD Tukey's test, pvalue < 0.05). No trend emerged from the pigment assays. These data were then integrated into the analyses designed to identify the driving forces behind the modifications of bacterial communities associated with the *Ulva* spp. surface.

Supplementary Table 2 : Details of algal surface (cm^2^) and moisture content (%). The surface area of each algal thallus was measured using the image analysis software ImageJ. Moisture content (%) was calculated as the percentage of weight loss after drying fresh algal biomass for 36 h at 60 °C.

| **Samples** | **Algal surface (cm²)** | **Moisture content** |
| --- | --- | --- |
| Fev21-S1-U1 | 20.6505 | 69.3877551 |
| Fev21-S1-U2 | 21.967 | 77.57009346 |
| Fev21-S1-U3 | 20.27066667 | 85.6 |
| Fev21-S2-U1 | 9.301 | 96.6442953 |
| Fev21-S2-U2 | 13.43316667 | 98.14814815 |
| Fev21-S2-U3 | 6.082166667 | 90.07633588 |
| Fev21-S3-U1 | 29.3905 | 95.26627219 |
| Fev21-S3-U2 | 27.40516667 | 75.78947368 |
| Fev21-S3-U3 | 16.14216667 | 86.08695652 |
| Avr21-S1-U1 | 164.459 | 78.60215054 |
| Avr21-S1-U2 | 113.2901667 | 85.52294101 |
| Avr21-S1-U3 | 115.3725 | 83.94294916 |
| Avr21-S2-U1 | 95.03033333 | 83.03780529 |
| Avr21-S2-U2 | 111.433 | 86.9 |
| Avr21-S2-U3 | 151.8886667 | 89.33803581 |
| Avr21-S3-U1 | 98.07816667 | 86.7896679 |
| Avr21-S3-U2 | 112.3976667 | 84.45626478 |
| Avr21-S3-U3 | 113.3661667 | 77.1040724 |
| Jun21-S1-U1 | 138.205 | 76.47058824 |
| Jun21-S1-U2 | 130.8586667 | 82.85714286 |
| Jun21-S1-U3 | 157.4945 | 81.81818182 |
| Jun21-S2-U1 | 153.5003333 | 85.71428571 |
| Jun21-S2-U2 | 108.6161667 | 88.88888889 |
| Jun21-S2-U3 | 82.1345 | 84.375 |
| Jun21-S3-U1 | 90.4845 | 84.90566038 |
| Jun21-S3-U2 | 92.0315 | 78.57142857 |
| Jun21-S3-U3 | 79.995 | 90.90909091 |
| Aou21-S1-U1 | 258.6945 | 81.63265306 |
| Aou21-S1-U2 | 188.3843333 | 83.33333333 |
| Aou21-S1-U3 | 175.3753333 | 87.17948718 |
| Aou21-S2-U1 | 180.0541667 | 85.96491228 |
| Aou21-S2-U2 | 189.7595 | 81.25 |
| Aou21-S2-U3 | 81.38416667 | 87.5 |
| Aou21-S3-U1 | 249.8691667 | 84.84848485 |
| Aou21-S3-U2 | 189.4046667 | 92.10526316 |
| Aou21-S3-U3 | 109.8908333 | 80 |
| Oct21-S1-U1 | 144.0233333 | 86.04651163 |
| Oct21-S1-U2 | 121.5411667 | 72.72727273 |
| Oct21-S1-U3 | 111.288 | 81.63265306 |
| Oct21-S2-U1 | 121.0123333 | 70 |
| Oct21-S2-U2 | 108.0536667 | 88 |
| Oct21-S2-U3 | 92.15 | 84.21052632 |
| Oct21-S3-U1 | 93.88833333 | 88.46153846 |
| Oct21-S3-U2 | 37.11566667 | 50 |
| Oct21-S3-U3 | 107.6641667 | 89.65517241 |
| Dec21-S1-U1 | 91.46233333 | 72.72727273 |
| Dec21-S1-U2 | 73.89 | 76.47058824 |
| Dec21-S1-U3 | 107.3613333 | 40 |
| Dec21-S2-U1 | 47.916 | 20 |
| Dec21-S2-U2 | 70.365 | 0 |
| Dec21-S2-U3 | 71.29033333 | 40 |
| Dec21-S3-U1 | 83.2665 | 84.21052632 |
| Dec21-S3-U2 | 61.83416667 | 85.71428571 |
| Dec21-S3-U3 | 93.48216667 | 88.88888889 |
| Fev22-S1-U1 | 61.20716667 | 90.47619048 |
| Fev22-S1-U2 | 91.9665 | 89.47368421 |
| Fev22-S1-U3 | 90.965 | 86.66666667 |
| Fev22-S2-U1 | 55.89133333 | 87.5 |
| Fev22-S2-U2 | 60.17516667 | 85.71428571 |
| Fev22-S2-U3 | 70.96216667 | 72.72727273 |
| Fev22-S3-U1 | 70.872 | 90.90909091 |
| Fev22-S3-U2 | 68.7385 | 90 |
| Fev22-S3-U3 | 48.78766667 | 85.18518519 |
| Avr22-S1-U1 | 93.5825 | 82.29166667 |
| Avr22-S1-U2 | 85.97716667 | 74.32432432 |
| Avr22-S1-U3 | 88.26783333 | 88.19188192 |
| Avr22-S2-U1 | 90.71183333 | 81.50289017 |
| Avr22-S2-U2 | 98.92083333 | 84.96732026 |
| Avr22-S2-U3 | 64.63333333 | 86.69950739 |
| Avr22-S3-U1 | 69.58383333 | 89.92443325 |
| Avr22-S3-U2 | 96.68916667 | 79.62962963 |
| Avr22-S3-U3 | 97.10916667 | 91.4893617 |
| Jun22-S1-U1 | 216.0053333 | 74 |
| Jun22-S1-U2 | 97.88083333 | 88 |
| Jun22-S1-U3 | 145.5526667 | 79 |
| Jun22-S2-U1 | 119.3083333 | 83 |
| Jun22-S2-U2 | 118.5333333 | 90 |
| Jun22-S2-U3 | 138.5658333 | 86 |
| Jun22-S3-U1 | 122.0046667 | 84 |
| Jun22-S3-U2 | 115.1401667 | 77 |
| Jun22-S3-U3 | 119.9293333 | 91 |
| Dec21-S1-U1 | 91.46233333 | 72.72727273 |
| Dec21-S1-U2 | 73.89 | 76.47058824 |
| Dec21-S1-U3 | 107.3613333 | 40 |
| Dec21-S2-U1 | 47.916 | 20 |
| Dec21-S2-U2 | 70.365 | 0 |
| Dec21-S2-U3 | 71.29033333 | 40 |
| Dec21-S3-U1 | 83.2665 | 84.21052632 |
| Dec21-S3-U2 | 61.83416667 | 85.71428571 |
| Dec21-S3-U3 | 93.48216667 | 88.88888889 |
| Fev22-S1-U1 | 61.20716667 | 90.47619048 |
| Fev22-S1-U2 | 91.9665 | 89.47368421 |
| Fev22-S1-U3 | 90.965 | 86.66666667 |
| Fev22-S2-U1 | 55.89133333 | 87.5 |
| Fev22-S2-U2 | 60.17516667 | 85.71428571 |
| Fev22-S2-U3 | 70.96216667 | 72.72727273 |
| Fev22-S3-U1 | 70.872 | 90.90909091 |
| Fev22-S3-U2 | 68.7385 | 90 |
| Fev22-S3-U3 | 48.78766667 | 85.18518519 |
| Avr22-S1-U1 | 93.5825 | 82.29166667 |
| Avr22-S1-U2 | 85.97716667 | 74.32432432 |
| Avr22-S1-U3 | 88.26783333 | 88.19188192 |
| Avr22-S2-U1 | 90.71183333 | 81.50289017 |
| Avr22-S2-U2 | 98.92083333 | 84.96732026 |
| Avr22-S2-U3 | 64.63333333 | 86.69950739 |
| Avr22-S3-U1 | 69.58383333 | 89.92443325 |
| Avr22-S3-U2 | 96.68916667 | 79.62962963 |
| Avr22-S3-U3 | 97.10916667 | 91.4893617 |
| Jun22-S1-U1 | 216.0053333 | 74 |
| Jun22-S1-U2 | 97.88083333 | 88 |
| Jun22-S1-U3 | 145.5526667 | 79 |
| Jun22-S2-U1 | 119.3083333 | 83 |
| Jun22-S2-U2 | 118.5333333 | 90 |
| Jun22-S2-U3 | 138.5658333 | 86 |
| Jun22-S3-U1 | 122.0046667 | 84 |
| Jun22-S3-U2 | 115.1401667 | 77 |
| Jun22-S3-U3 | 119.9293333 | 91 |

## **Supplementary information for characterization of the environment**

Supplementary Table 3 : Environmental parameters measured in situ using a multiparameter probe (YSI 6920 V2-2, A xylem brand)

| Date | Station | pH | Chl a (µg/L) | Water depth (m) | Temperature (°C) | Turbidity (NTU) | Salinity | Dissolved oxygen (mg/L) |
| --- | --- | --- | --- | --- | --- | --- | --- | --- |
| 02/12/2021 | 1 | 7.9 | ND | 0.3 | 9.4 | 9.6 | ND | 10.3 |
|  | 2 | 7.9 | ND | 0.2 | 7.9 | 0.2 | ND | 11.1 |
|  | 3 | 7.9 | ND | 0.4 | 8.5 | 0 | ND | 11.6 |
| 28/06/2022 | 1 | 8.4 | ND | 0.95 | 18.46 | ND | 34.38 | 7.57 |
|  | 2 | 8.8 | ND | 0.61 | 18.51 | 4.6 | 34.44 | 12.6 |
|  | 3 | 8.7 | ND | 0.6 | 18.92 | 0.5 | 34.55 | 13.05 |

Supplementary Table 4 : Environmental parameters used for the envfit analyses; fuchsia data were retrieved from Previmer models, while turquoise data originated from Météo-France.

| **Sample date** | **February 2021** | **April 2021** | **June 2021** | **August 2021** | **October 2021** |
| --- | --- | --- | --- | --- | --- |
| **Sea surface temp.** | 8.446428571 | 10.775 | 16.3 | 17.8 | 15.4 |
| **Salinity** | 32.38571429 | 34,0 | 34.6 | 35.4 | 34.8 |
| **Chlorophyll a** | 0.3877777778 | 1.744333333 | 1.9463 | 3.194 | 1.49 |
| **Nitrates** | 20.46785714 | 7.966 | 0.62496 | 2.24 | 1.03 |
| **Phosphate** | 0.4745 | 0.1161666667 | 0.08074333333 | 0.26 | 0.22 |
| **Temp. mean** | 7.471428571 | 9.796666667 | 16.06333333 | 16.6483871 | 13.41290323 |
| **Temp. min** | 4.942857143 | 4.523333333 | 12,0 | 13.1 | 10,0 |
| **Temp. max** | 10.6 | 15.6 | 21,0 | 21.3 | 17.8 |
| **Wind speed** | 1.946428571 | 1.738709677 | 1.213333333 | 1.341935484 | 1.174193548 |
| **Relative humidity** | 85.85714286 | 66.03333333 | 82.8666666666667 | 83.3870967741936 | 87.6129032258064 |
| **Radiation** | 577.321428571429 | 1856.4 | 1794.8 | 1683.35483870968 | 879.838709677419 |
| **Insolation time** | 188.035714285714 | 518.8 | 341.433333333333 | 377.903225806452 | 306.193548387097 |
| **Precipitations** | 3.53214286 | 0.65 | 1.6 | 1.48709677419355 | 4.80967741935484 |

| **Sample date** | **December 2021** | **February 2022** | **April 2022** | **June 2022** |
| --- | --- | --- | --- | --- |
| **Sea surface temp.** | 10.6 | 9.3 | 11.31666667 | 16.4 |
| **Salinity** | 35.1 | 34.1 | 34.06666667 | 34.6 |
| **Chlorophyll a** | 0.53 | 0.38 | 2.106666667 | 2.805333333 |
| **Nitrates** | 4.63 | 15.91 | 14.122 | 3.018333333 |
| **Phosphate** | 0.3868387097 | 0.45675 | 0.2238133333 | 0.1027166667 |
| **Temp. mean** | 8.561290323 | 8.503571429 | 11.1 | 16.73333333 |
| **Temp. min** | 5.8 | 5.5 | 6.6 | 12.1 |
| **Temp. max** | 11.6 | 11.8 | 16.5 | 22.1 |
| **Wind speed** | 1.75483871 | 1.842857143 | 1.57 | 1.31 |
| **Relative humidity** | 90.7096774193548 | 87.6071428571429 | 76.0666666666667 | 77.6333333333333 |
| **Radiation** | 257.870967741935 | 589.928571428571 | 1755.23333333333 | 2062.83333333333 |
| **Insolation time** | 107.58064516129 | 163.642857142857 | 441.4 | 457.566666666667 |
| **Precipitations** | 3.94838709677419 | 3.1 | 1.58333333333333 | 3.00666666666667 |

## **Supplementary information for microbiota analysis**


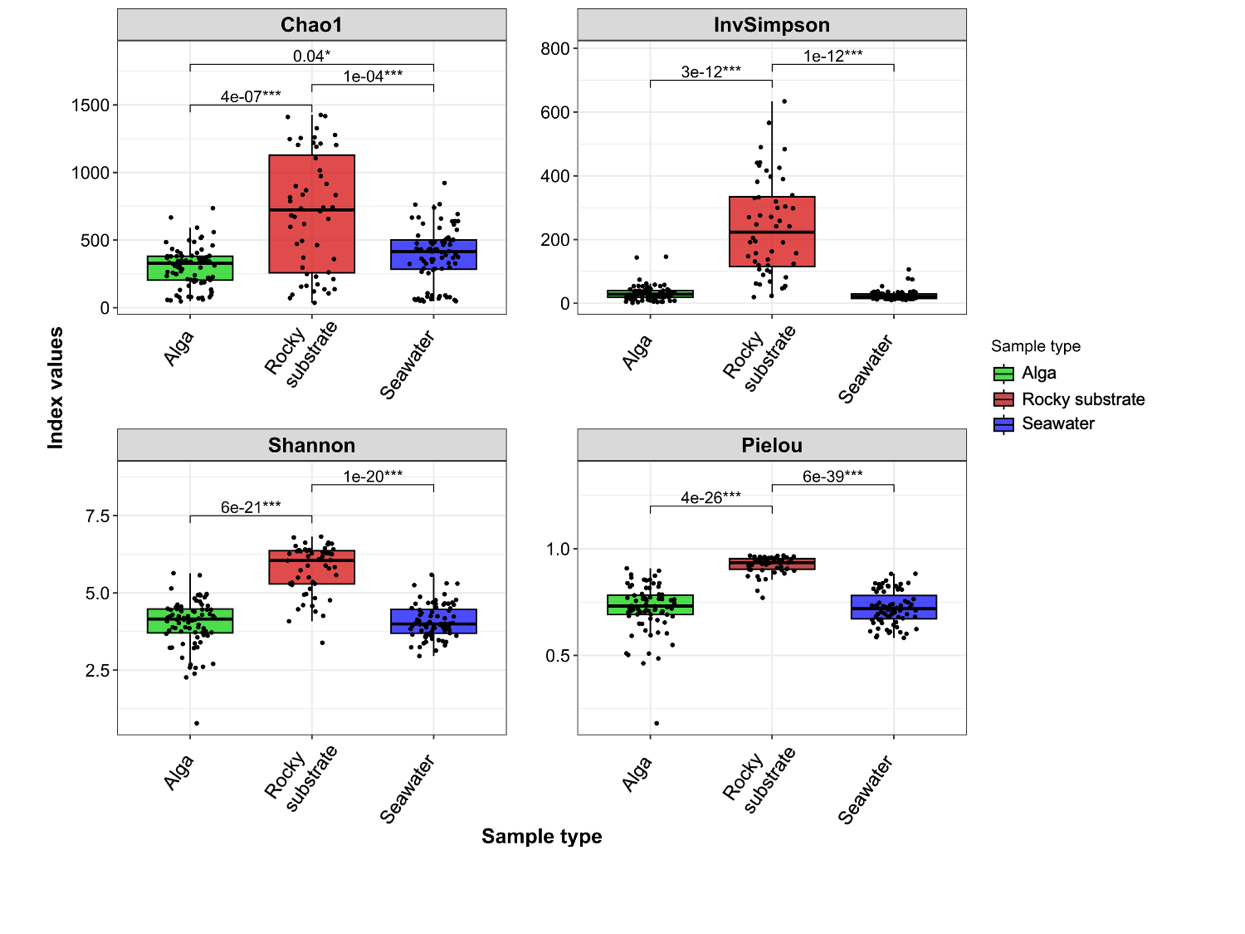


Supplementary Figure 5 : Alpha-diversity of bacterial community associated with the three habitats: Ulva spp. (green), rocky substrate (red) and seawater (blue) (p < 0.05 [T-test]).


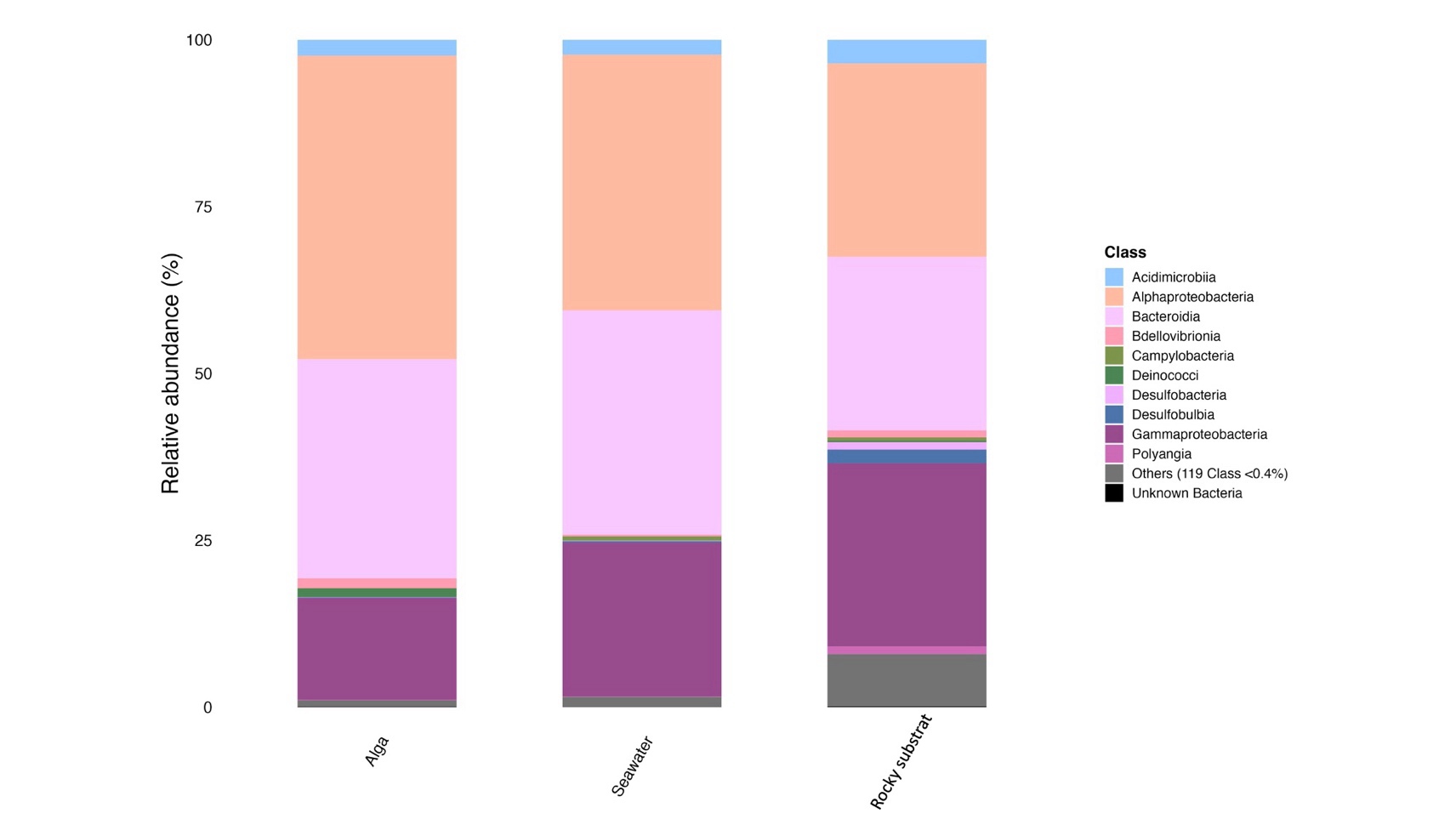


Supplementary Figure 6 : Relative abundance of majoritarian bacterial ASVs summarized at the class taxonomic rank in associated communities of Ulva spp., seawater and rocky substrate


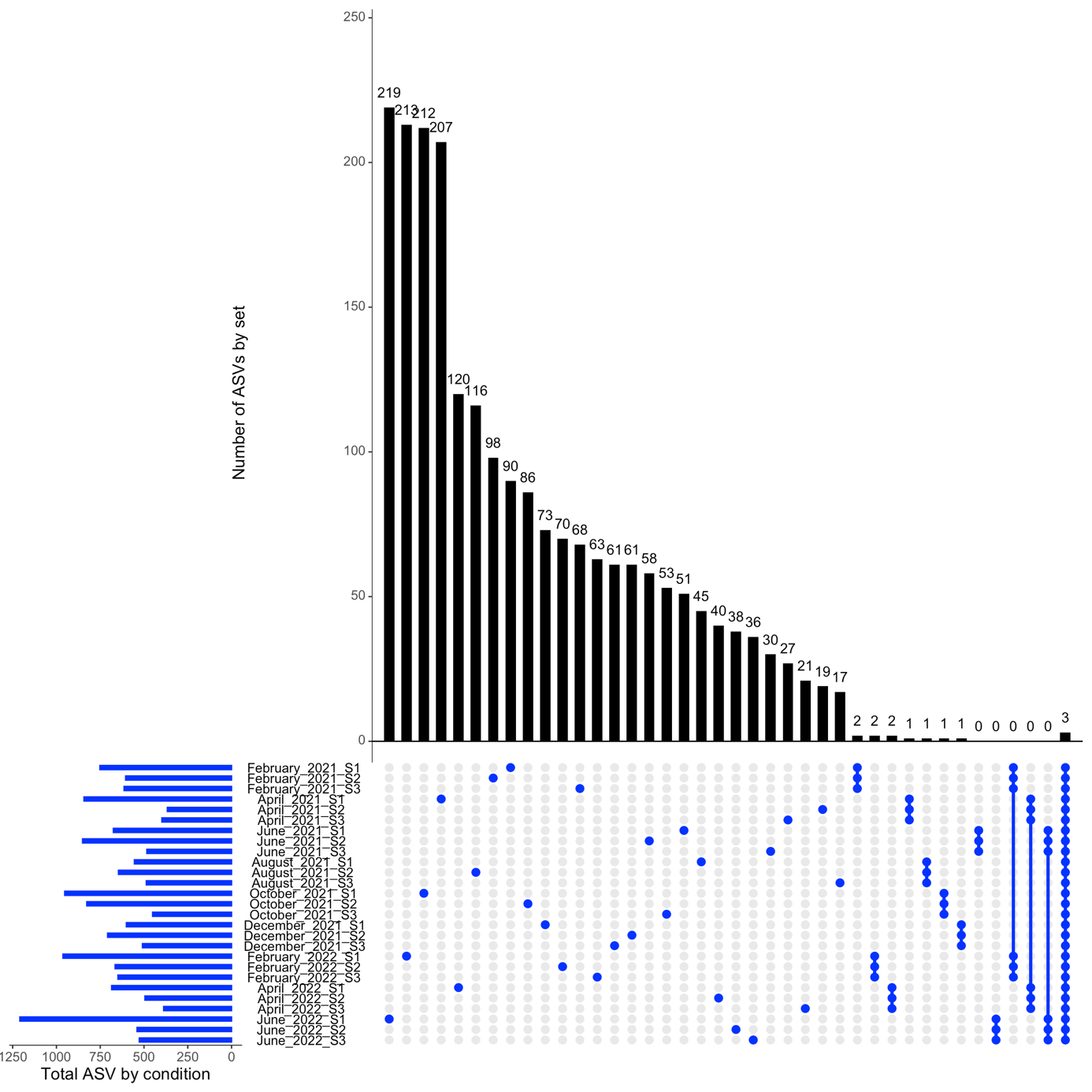


Supplementary Figure 7 : Venn diagram of ASVs by sampling date and station

**
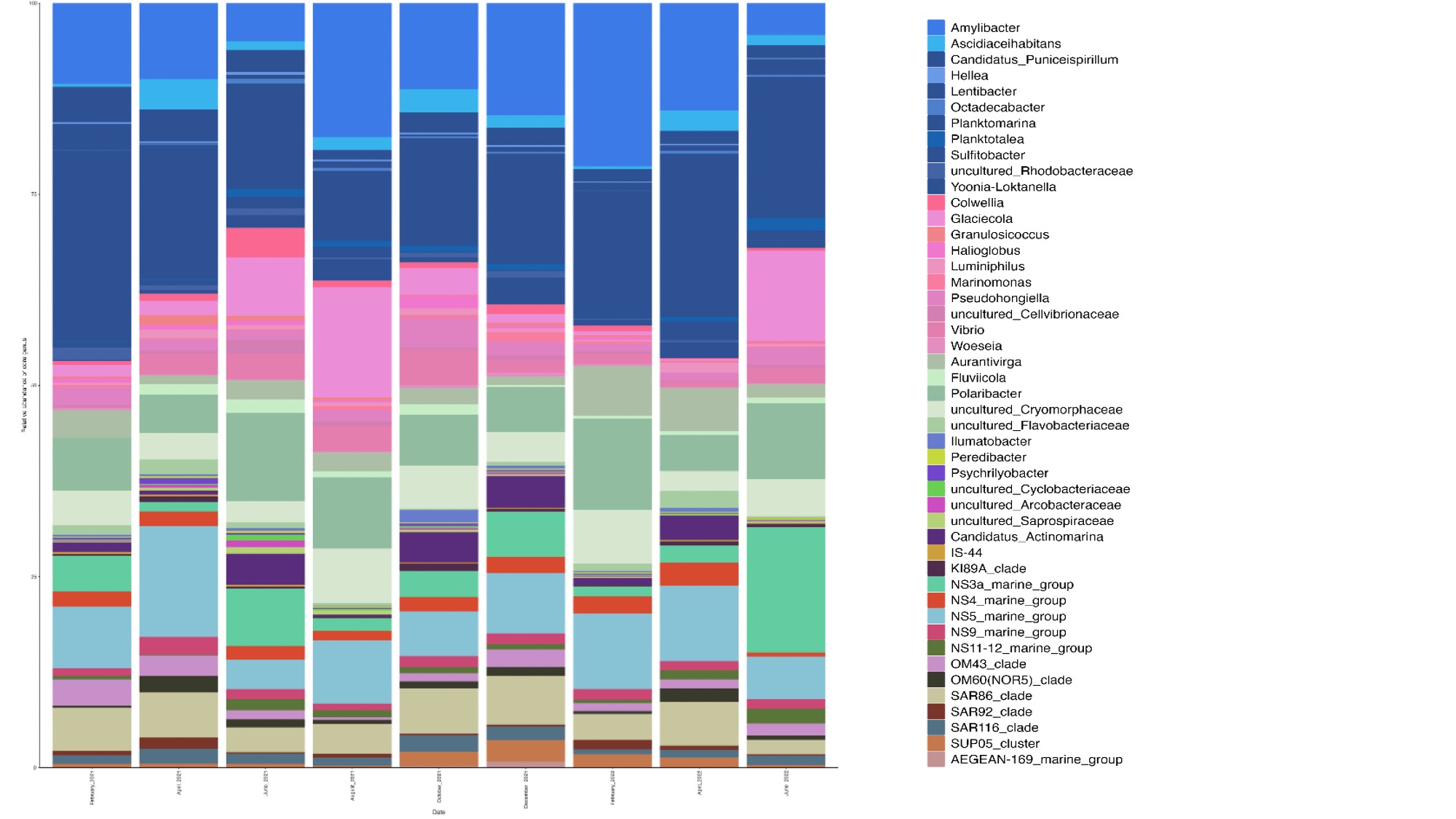
**

Supplementary Figure 8 : Relative abundance of ASVs belonging to the stable seawater bacterial community, summarized at the genera taxonomic rank

## **Supplementary information for multiomics analysis**


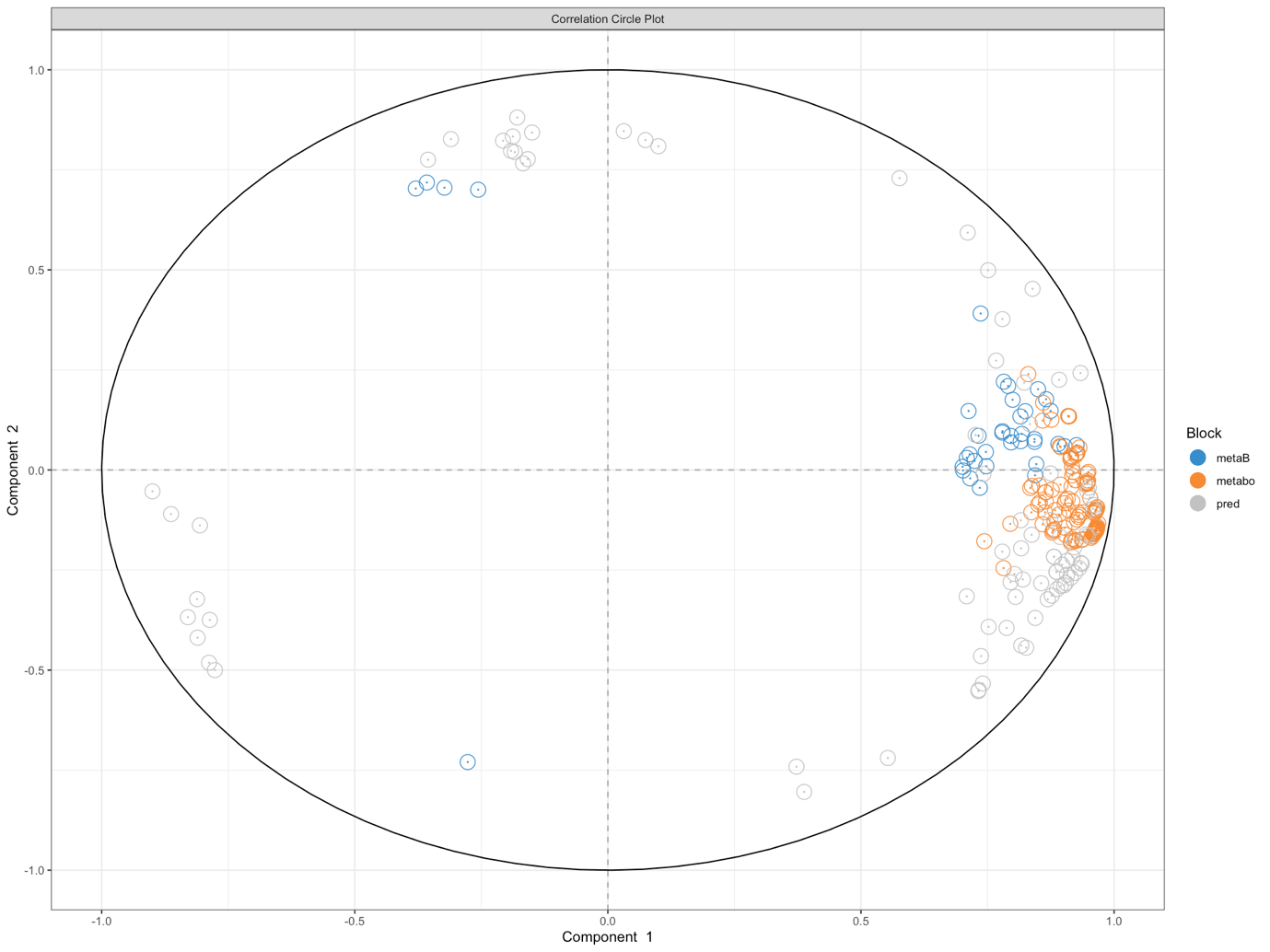


Supplementary Figure 9 : Correlation of ASVs relative abundances (blue), chemical features (orange) and predictive cellular function (grey) datasets
